# VipD of *Legionella pneumophila* Targets Activated Rab5 and Rab22 to Interfere with Endosomal Trafficking in Macrophages

**DOI:** 10.1371/journal.ppat.1003082

**Published:** 2012-12-13

**Authors:** Bonsu Ku, Kwang-Hoon Lee, Wei Sun Park, Chul-Su Yang, Jianning Ge, Seong-Gyu Lee, Sun-Shin Cha, Feng Shao, Won Do Heo, Jae U. Jung, Byung-Ha Oh

**Affiliations:** 1 Department of Biological Sciences, KAIST Institute for the Biocentury, Korea Advanced Institute of Science and Technology, Daejeon, Korea; 2 Department of Molecular Microbiology and Immunology, Keck School of Medicine, University of Southern California, Los Angeles, California, United States of America; 3 National Institute of Biological Sciences, Beijing, China; 4 Marine Biotechnology Research Center, Korea Ocean Research and Development Institute, Ansan, Korea; Osaka University, Japan

## Abstract

Upon phagocytosis, *Legionella pneumophila* translocates numerous effector proteins into host cells to perturb cellular metabolism and immunity, ultimately establishing intracellular survival and growth. VipD of *L. pneumophila* belongs to a family of bacterial effectors that contain the N-terminal lipase domain and the C-terminal domain with an unknown function. We report the crystal structure of VipD and show that its C-terminal domain robustly interferes with endosomal trafficking through tight and selective interactions with Rab5 and Rab22. This domain, which is not significantly similar to any known protein structure, potently interacts with the GTP-bound active form of the two Rabs by recognizing a hydrophobic triad conserved in Rabs. These interactions prevent Rab5 and Rab22 from binding to downstream effectors Rabaptin-5, Rabenosyn-5 and EEA1, consequently blocking endosomal trafficking and subsequent lysosomal degradation of endocytic materials in macrophage cells. Together, this work reveals endosomal trafficking as a target of *L. pneumophila* and delineates the underlying molecular mechanism.

## Author Summary

*Legionella pneumophila* is a pathogen bacterium that causes Legionnaires' disease accompanied by severe pneumonia. Surprisingly, this pathogen invades and replicates inside macrophages, whose major function is to detect and destroy invading microorganisms. How *L. pneumophila* can be “immune” to this primary immune cell has been a focus of intensive research. Upon being engulfed by a macrophage cell, *L. pneumophila* translocates hundreds of bacterial proteins into this host cell. These proteins, called bacterial effectors, are thought to manipulate normal host cellular processes. However, which host molecules and how they are targeted by the bacterial effectors are largely unknown. In this study, we determined the three-dimensional structure of *L. pneumophila* effector protein VipD, whose function in macrophage was unknown. Ensuing analyses revealed that VipD selectively and tightly binds two host signaling proteins Rab5 and Rab22, which are key regulators of early endosomal vesicle trafficking. These interactions prevent the activated form of Rab5 and Rab22 from binding their downstream signaling proteins, resulting in the blockade of endosomal trafficking in macrophages. The presented work shows that *L. pneumophila* targets endosomal Rab proteins and delineates the underlying molecular mechanism, providing a new insight into the pathogen's strategies to dysregulate normal intracellular processes.

## Introduction

*Legionella pneumophila* is an opportunistic human pathogen that replicates inside macrophages, which are at the front line of immune defense. This Gram-negative bacterium causes Legionnaires' disease characterized by severe pneumonia or less acute Pontiac fever. By phagocytosis, the bacteria are enclosed in a membrane-bound vacuole, called *Legionella*-containing vesicle (LCV). This vesicle evades the endocytic pathway to avoid fusion with lysosomes [1], and becomes the growth and replication niche for the bacteria [2], [3]. The intracellular survival and replication depend on the Dot/Icm type IV secretion system of the bacterium, which translocates about 270 effector proteins into the host cytosol [4], [5]. Understanding of detailed molecular mechanisms of the *L. pneumophila* effectors has been achieved for a number of proteins, including SidM (substrate of Icm/Dot transporter M; also known as DrrA) [6]–[12], LpGT (*L. pneumophila* glucosyltransferase; also known as Lgt1) [13]–[15], AnkX (Ankyrin repeat protein X) [16]–[18] and others as reviewed recently [19].

VipD (*v*acuolar protein sorting *i*nhibitor *p*rotein *D*) is one of the *L. pneumophila* effector proteins, which interrupts Golgi-to-vacuole trafficking of three yeast proteins (carboxypeptidase S, carboxypeptidase Y and alkaline phosphatase) as well as endoplasmic reticulum (ER)-to-Golgi trafficking of carboxypeptidase Y when expressed in *Saccharomyces cerevisiae* [20]. VipD contains an N-terminal lipase domain which shares sequence homology with patatin, a phospholipase in potato tuber having phospholipase A and lysophospholipase A activities [21]. A similar lipase domain is present in two other *L. pneumophila* effector proteins VpdA and VpdB [22] and in ExoU of *Pseudomonas aeruginosa*, which is a potent secreted cytotoxin [23], [24]. On the other hand, their C-terminal domains do not exhibit sequence homology with each other or with any functionally annotated protein domain. Previously, overexpression of VipD was shown to be mildly toxic to 293T cells and *S. cerevisiae*, and its toxicity was only partially dependent on the putative lipase activity of the protein [22]. Moreover, a VipD fragment lacking the N-terminal lipase domain interfered with vesicle transport in *S. cerevisiae* to a much greater extent than full-length VipD did [20], indicating that the C-terminal domain is critical for the function of VipD. But, how the C-terminal domain of VipD perturbs vesicle trafficking in yeast is unknown. It is also unknown whether VipD may manipulate intracellular trafficking in macrophages, the major mammalian host cells of *L. pneumophila*.

We undertook an integrative approach involving X-ray crystallography, biochemistry and cellular imaging to understand whether and how VipD might affect mammalian host cells. We show that the C-terminal domain of VipD tightly binds to the GTP-bound form of Rab5 and Rab22, blocks their interactions with three downstream effector molecules, and inhibits endocytic trafficking in mouse macrophages. Together, this study demonstrates that VipD targets and interferes with endosomal membrane trafficking in mammalian host cells.

## Results

### VipD adopts a two-domain fold

**Figure 1:**
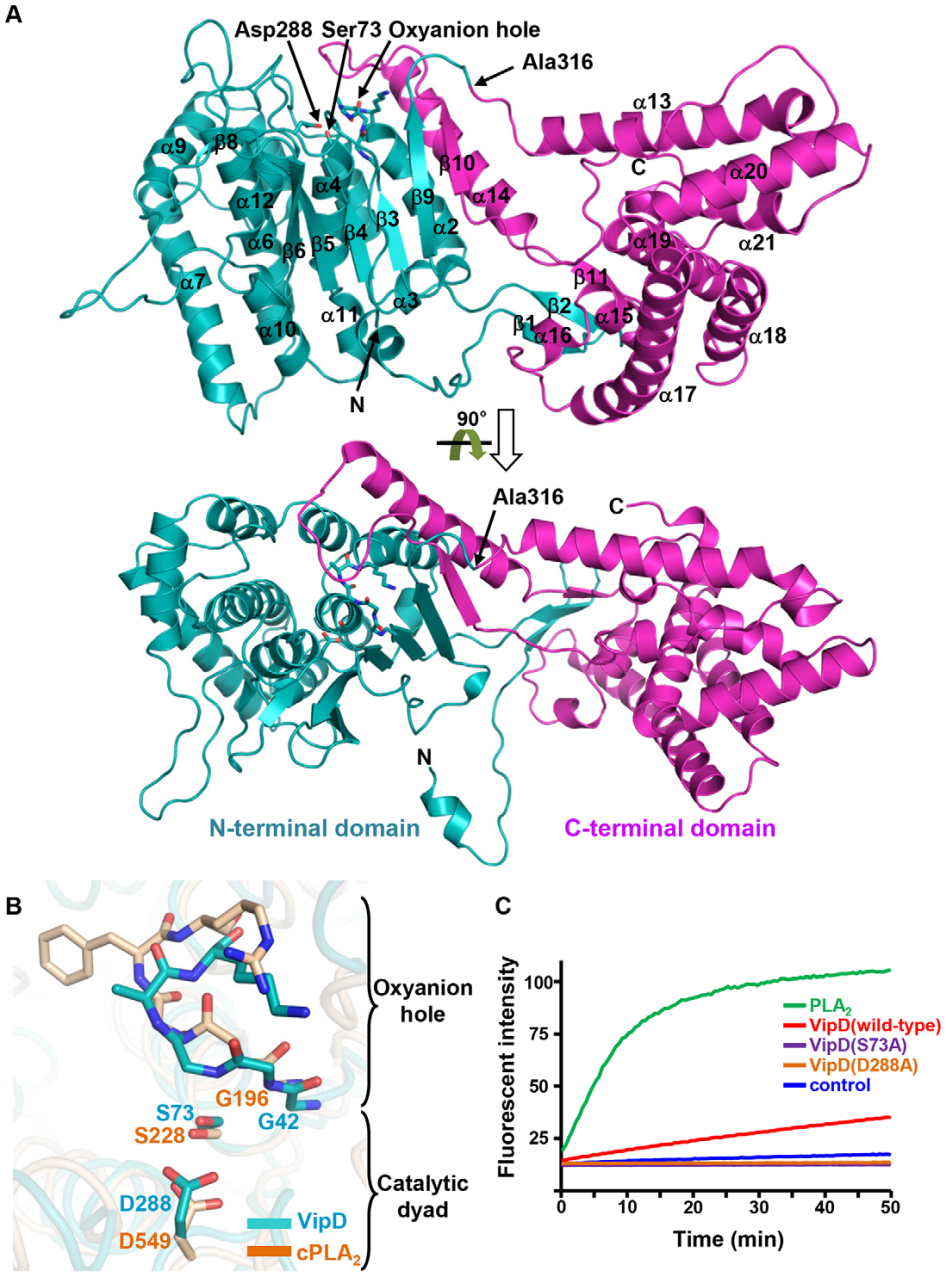
Structural features of VipD. (A) Two views of overall structure. The two domains delimited by Ala316 are in two different colors. The sticks representation highlights the positions of the catalytic dyad (Ser73 and Asp288) and the oxyanion hole-forming residues (Gly42-Gly-Gly-Ala-Lys46). (B) Comparison of the catalytic motifs between VipD and cPLA_2_. The structures of VipD and cPLA_2_ were superposed and the catalytic dyad and the oxyanion hole-forming residues are highlighted by stick presentation. Their spatial positions in the two structures are closely similar. (C) Phospholipase activity. The phospholipase A_2_ activity was measured using the EnzChek PLA_2_ assay kit in the presence of VipD (wild-type, S73A or D288A) (10 µM) or phospholipase A_2_ from honey bee venom (5 units/mL).

Full-length VipD was crystallizable, but the X-ray diffraction of the crystals was too poor for structure determination. Various attempts were made to improve the crystal quality, and the successful trial was to employ a truncated VipD lacking C-terminal 46 residues and to dehydrate resulting crystals with 30% glycerol at −20°C. The structure of this truncated version of VipD, referred to as VipD(1-575), was determined at 2.9 Å resolution (Table 1). VipD(1-575) folds into two domains which are roughly discernable: the N- and C-terminal domains, designated as VipD(1-316) and VipD(316-575), respectively (Figure 1A). Ala316 is at the boundary of the two domains and located in the middle of the structure lengthwise (Figure 1A). The two domains interact with each other mostly through secondary structural elements. β1 and β2 of VipD(1-316) form a “mini” β-sheet together with β11 of VipD(316-575) in the C-terminal domain. Likewise, β10 of VipD(316-575) is a part of the central β-sheet in the N-terminal domain. In addition, α14 of VipD(316-575) interacts with α2 of VipD(1-316) (Figure 1A). These observations suggested that division of VipD into the two fragments containing residues 1-316 or 316-575 would result in misfolded proteins. However, both VipD(1-316) and VipD(316-575) or VipD(316-621) produced in *E. coli* were soluble and purifiable.

**Table 1:** Data collection and structure refinement statistics.

	Native	Se-Met
Space group	*I*432	*I*432
Unit cell dimensions		
a, b, c (Å)	252.76, 252.76, 252.76	253.35, 253.35, 253.35
α, β, γ (°)	90, 90, 90	90, 90, 90
Wavelength (Å)	1.0000	0.9795 (*peak*)
Resolution (Å)	50.0 - 2.8 (2.85 - 2.80)^a^	50.0 - 3.3 (3.36 - 3.30)
*R*_sym_ b	8.9 (45.9)	12.3 (25.3)
*I*/σ(*I*)	18.5 (2.3)	14.4 (2.2)
Completeness (%)	99.4 (98.9)	94.6 (74.9)
Redundancy	9.5	11.4
Refinement		
Resolution (Å)	30.0 - 2.9	
Number of reflections	30527	
*R*_work_ c/*R*_free_	21.4/23.3	
Number of atoms		
Protein		
Protein	4380	
Water	86	
R.m.s deviations		
Bond lengths (Å)	0.009	
Bond angles (°)	1.481	
Ramachandran plot (%)		
Most favored region	90.7	
Additionally allowed region	9.3	
Average B-values (Å^2^)		
Protein	68.1	
Water	47.4	

a The numbers in parentheses are statistics from the highest resolution shell.

b *R*_sym_ = Σ|*I*_obs_−*I*_avg_|/*I*_obs_, where *I*_obs_ is the observed intensity of individual reflection and *I*_avg_ is average over symmetry equivalents.

c *R*_work_ = Σ||*F*_o_|−|*F*_c_||/Σ|*F*_o_|, where |*F*_o_| and |*F*_c_| are the observed and calculated structure factor amplitudes, respectively. *R*_free_ was calculated with 5% of the data.

A search for similar structures in the Protein Data Bank with the program Dali [25] showed that the N-terminal domain is most homologous to patatin (PDB entry: 1OXW) and cytosolic phospholipase A_2_ (cPLA_2_; PDB entry: 1CJY) with the Z-scores of 14.0 and 11.4, respectively (Figure S1A). In particular, the two residues of cPLA_2_ (Ser228 and Asp549), which form the catalytic dyad [26], are closely superposable on Ser73 and Asp288 in VipD (Figure 1B). Moreover, the Gly196-Gly-Gly-Phe-Arg200 sequence, which forms the oxyanion hole in cPLA_2_, is also present in VipD as a Gly42-Gly-Gly-Ala-Lys46 sequence at spatially the same location (Figure 1B). In cPLA_2_, the active site groove containing the catalytic dyad is partially covered by loop αH-αI. Likewise, a similar groove covered by loop β10-α14 is present in VipD (Figure S2). These features indicate that VipD is a catalytically active phospholipase A_2_. However, whether VipD has an intrinsic phospholipase A_2_ activity or not has been unsettled [22], [27]. We examined a phospholipase A_2_ activity of VipD by using an artificial fluorogenic phospholipid substrate red/green BODIPY PC-A2 (specific for PLA_2_ enzyme), and show here that VipD has a phospholipase A_2_ activity (Figure 1C). Alanine substitution of Ser73 or Asp288 abrogated the lipase activity of VipD, demonstrating that the two residues indeed form a catalytic dyad (Figure 1C).

VipD(316-575) contains ten α-helices and two short β-strands. This domain is not obviously homologous to any of the known protein structures in the Protein Data Bank. The best match (Z-score: 4.6) in the Dali search was the structure of the Vps9 domain of Rabex-5 (PDB entry: 1TXU), which is a guanine nucleotide exchange factor (GEF) for Rab5, Rab21 and Rab22 [28], [29]. Superposition of the two structures showed only a gross similarity in the spatial arrangement of five out of ten α-helices in VipD(316-575) (Figure S1B), providing only an unconvincing clue for the function of the C-terminal domain.

### VipD localizes to early endosomes via the C-terminal domain

**Figure 2:**
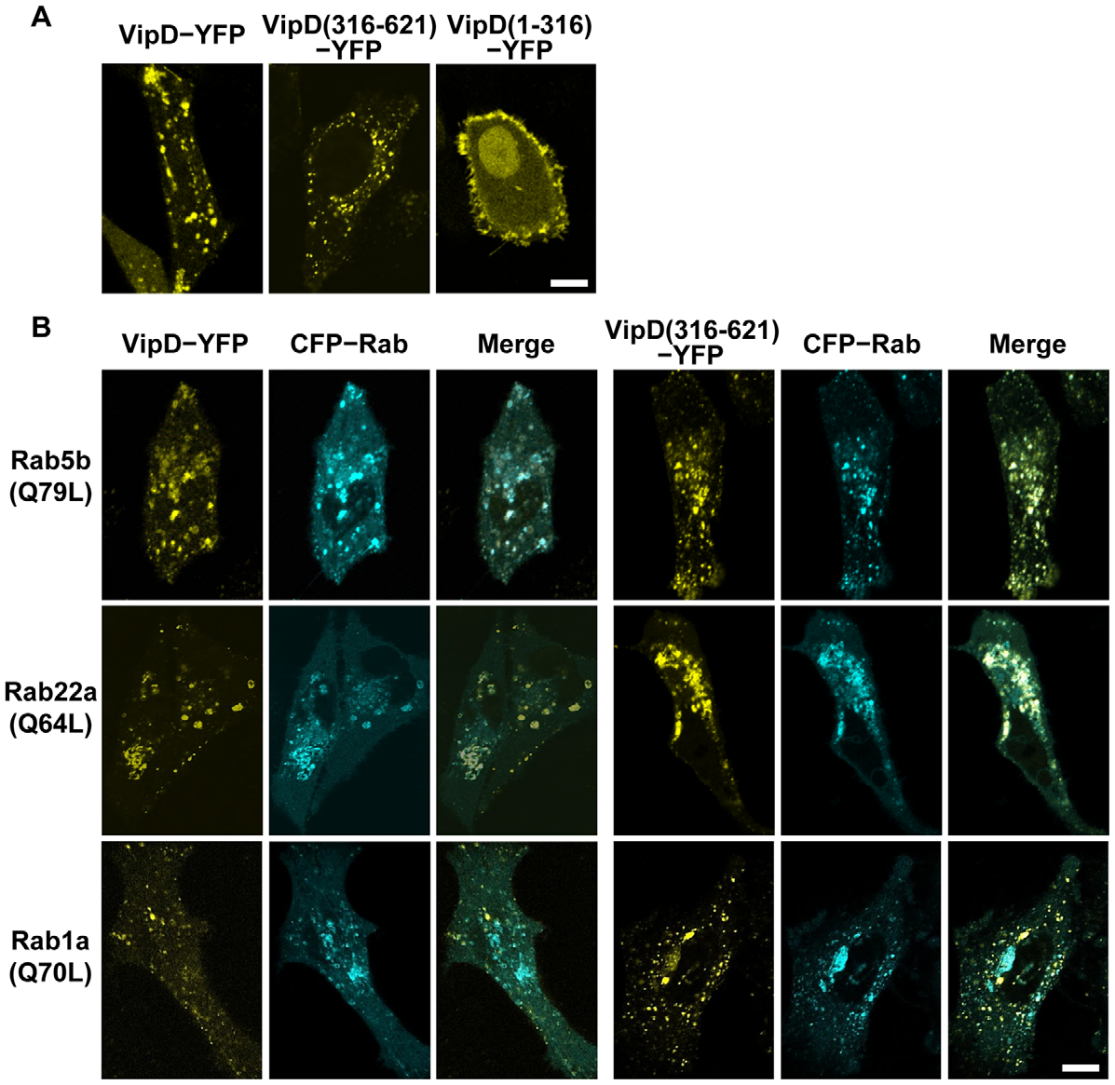
VipD colocalizes with Rab5b and Rab22a to early endosomes via the C-terminal domain. Shown are the confocal images of HeLa cells transiently expressing YFP-tagged VipD proteins and CFP-tagged Rab proteins. The scale bars indicate 10 µm. (A) The subcellular localization of VipD(1-316) is different from that of full-length VipD and VipD(316-621). (B) Full-length VipD colocalized with Rab5b(Q79L) or Rab22a(Q64L) but not with Rab1a(Q70L) when coexpressed together. Virtually the same colocalization was observed with VipD(316-621).

A clue for the biochemical function of the C-terminal domain of VipD was obtained by investigating the subcellular localization of VipD. In HeLa cells, full-length VipD, VipD(1-316) or VipD(316-621) was transiently expressed, each as a fusion protein containing yellow fluorescent protein (YFP) at the C-terminus. Full-length VipD and VipD(316-621) exhibited a similar fluorescence pattern, which was indicative of endosomal localization (Figure 2A). To elaborate this observation further, full-length VipD or VipD(316-621) was coexpressed with the early endosomal markers Rab5b and Rab22a and also with the ER-to-Golgi trafficking regulator Rab1a [30], respectively, in HeLa cells and in RAW264.7 macrophages. The GTPase-defective constitutively active forms, Rab5b(Q79L), Rab22a(Q64L) and Rab1a(Q70L), were employed, all tagged with cyan fluorescent protein (CFP). Both full-length VipD and VipD(316-621) colocalized with Rab5b(Q79L) and Rab22a(Q64L), but not with Rab1a(Q70L), in both types of cells (Figures 2B and S3). In contrast, VipD(1-316) was evenly dispersed throughout cells with a noticeable enrichment at the plasma membranes (Figure 2A). Notably, the characteristic tubular structures of endosomes observed with the expression of Rab22a(Q64L) alone (Figure S4A) [31] disappeared when this Rab protein was coexpressed together with full-length VipD or VipD(316-621), while their formation was unaffected by the expression of VipD(1-316) (Figure S4B). We additionally noted that Rab22a(Q64L) colocalized with Rab5b(Q79L) without inducing the tubular structures when the two proteins were coexpressed in both types of cells (Not shown). We also found that VipD colocalized with the wild-type forms of Rab5b and Rab22a (Figure S5A), which cycle between the endosomal membrane and the cytosol [30]. Finally, like Rab5b(Q79L), full-length VipD did not localize to lysosomes, as probed by the lysosomal marker Lysotracker Red (Figure S5B). These results convincingly indicated that VipD localizes to early endosomes via the C-terminal domain of the protein. In addition, the precise overlaps of the two different fluorescence images suggested that the C-terminal domain of VipD may directly interact with the two Rabs.

### VipD tightly binds to specific endosomal Rabs

**Figure 3:**
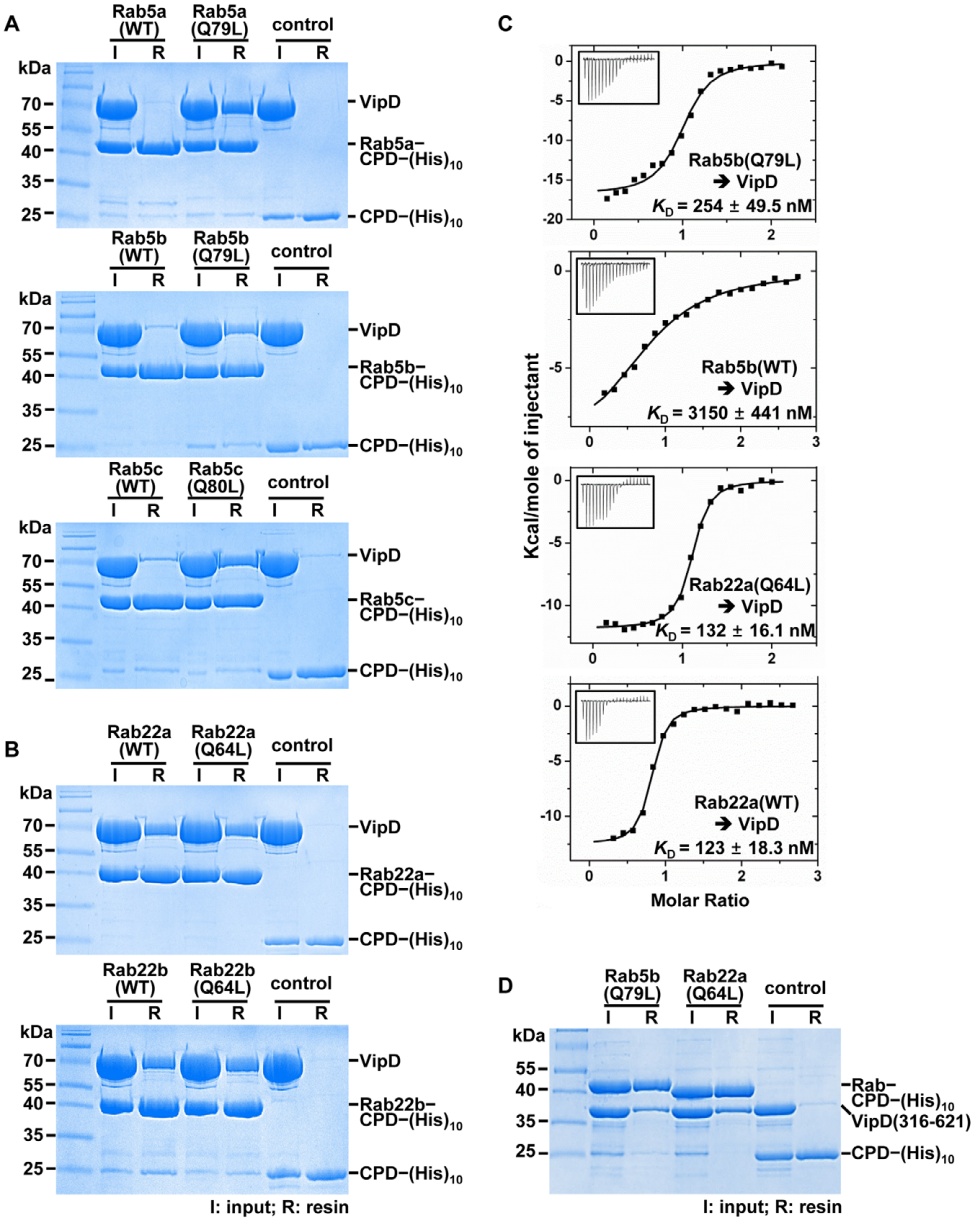
Interaction of VipD with Rab5 and Rab22. (A–B) (His)_10_ pull-down assay with full-length VipD. The indicated Rab proteins fused to CPD–(His)_10_ were incubated with VipD and Co^2+^ resin. Both the wild-type (WT) and the Q-to-L mutant Rabs were tested. Input proteins (I) and Co^2+^ resin-bound proteins (R) were visualized on a denaturing gel. VipD bound to the active forms of Rab5a, Rab5b and Rab5c preferentially over the inactive forms of the three Rabs (*A*; lanes 2 to 5). VipD bound tightly to both the active and the inactive forms of the two isoforms of Rab22 (*B*; lanes 2 to 5). VipD did not interact with CPD–(His)_10_ (*A* and *B*; control lanes). (C) ITC analysis. The measurement was carried out by titrating 0.2 mM of the indicated Rab proteins into 20 µM of VipD. The *K*_D_ values were deduced from curve fittings of the integrated heat per mole of added ligand. (D) (His)_10_ pull-down assay with VipD(316-621). VipD(316-621) was coprecipitated with the active form of Rab5b and Rab22a similarly as full-length VipD (*A* and *B*).

The possibility of the direct interactions of VipD with Rab5b and Rab22a was probed using Rab5b(1-190;Q79L) and Rab22a(1-175;Q64L), and a wild-type version of the two Rabs. These Rab proteins were C-terminally fused to a (His)_10_-tagged cysteine protease domain (CPD) to improve the solubility of the target proteins [32]. Indeed, VipD interacted with the GTP-bound Q-to-L mutant form of the two Rabs in a (His)_10_ pull-down assay (Figures 3A, second panel and 3B, first panel; lane 5). VipD also interacted with the wild-type version of the two Rabs in the GDP-bound inactive form, although its interaction with Rab5b(1-190):GDP was comparatively quite weak (Figures 3A, second panel and 3B, first panel; lane 3). Quantification of these interactions by isothermal titration calorimetry (ITC) revealed that VipD bound tightly to Rab5b(1-190;Q79L):GTP, but weakly to Rab5b(1-190):GDP, with the dissociation constants (*K*_D_) of 254 nM and 3150 nM, respectively (Figure 3C). In comparison, VipD interacted with both the GTP-bound and the GDP-bound forms of Rab22a(1-175) tightly with the similar *K*_D_ values of 132 nM and 123 nM, respectively (Figure 3C). The binding interactions are through the C-terminal domain of VipD, because VipD(316-621) interacted with Rab5b(1-190;Q79L):GTP and Rab22a(1-175;Q64L):GTP similarly as full-length VipD (Figure 3D). The (His)_10_ pull-down assay was also performed with Rab5a and Rab5c, the two other isoforms of Rab5. VipD bound to the two forms of Rab5a and Rab5c similarly as it did to Rab5b: tightly to the active form and weakly to the inactive form (Figure 3A; first and third panels). We also examined the interaction between VipD and Rab22b (also known as Rab31), the other isoform of Rab22. VipD tightly bound to both the GTP-bound and the GDP-bound forms of Rab22b(1-175), as it did to the two forms of Rab22a(1-175) (Figure 3B; second panel). We, in turn, examined whether these isoforms of Rab5 and Rab22 colocalize with VipD in HeLa cells. The active forms of Rab5a and Rab5c indeed colocalized with VipD, as Rab5b(Q79L) did (Figure S5C). However, Rab22b(Q64L) did not colocalize with VipD (Figure S5D). Rab22b is known to be largely associated with the trans-Golgi network in HeLa cells [33]. Consistently, the subcellular distribution of Rab22b(Q64L) overlapped only partially with that of the endosomal marker Rab22a(Q64L) (Figure S5D). These observations implied that the endosomal localization of VipD does not simply depend on the interaction with Rab proteins. To test this notion, a set of small interfering RNAs (siRNAs) was prepared which blocked the expression of Rab5a, Rab5b, Rab5c and Rab22a [31], [34]–[36]. Treatment of HeLa cells with each siRNA alone or together did not block the endosomal localization of VipD (Figure S6), indicating that VipD localizes to endosomes through an as yet unknown mechanism and then interacts with the endosomal Rab proteins.

To learn whether VipD might interact with other Rabs that are known to mediate endosomal trafficking, wild-type and GTPase-defective versions of Rab4b(1-178), Rab7a(1-190), Rab9a(1-185), Rab14(1-189) and Rab21(15-200) were produced. Additionally, we also produced two other Rabs, Rab1a(1-182) and Rab2a(1-182), which mediate trafficking between ER and Golgi. In the (His)_10_ pull-down assay, VipD did not exhibit a noticeable interaction with both the active and the inactive forms of all the seven Rab proteins (Figure S7).

### VipD is a very weak GEF antagonist and not a GAP

**Figure 4:**
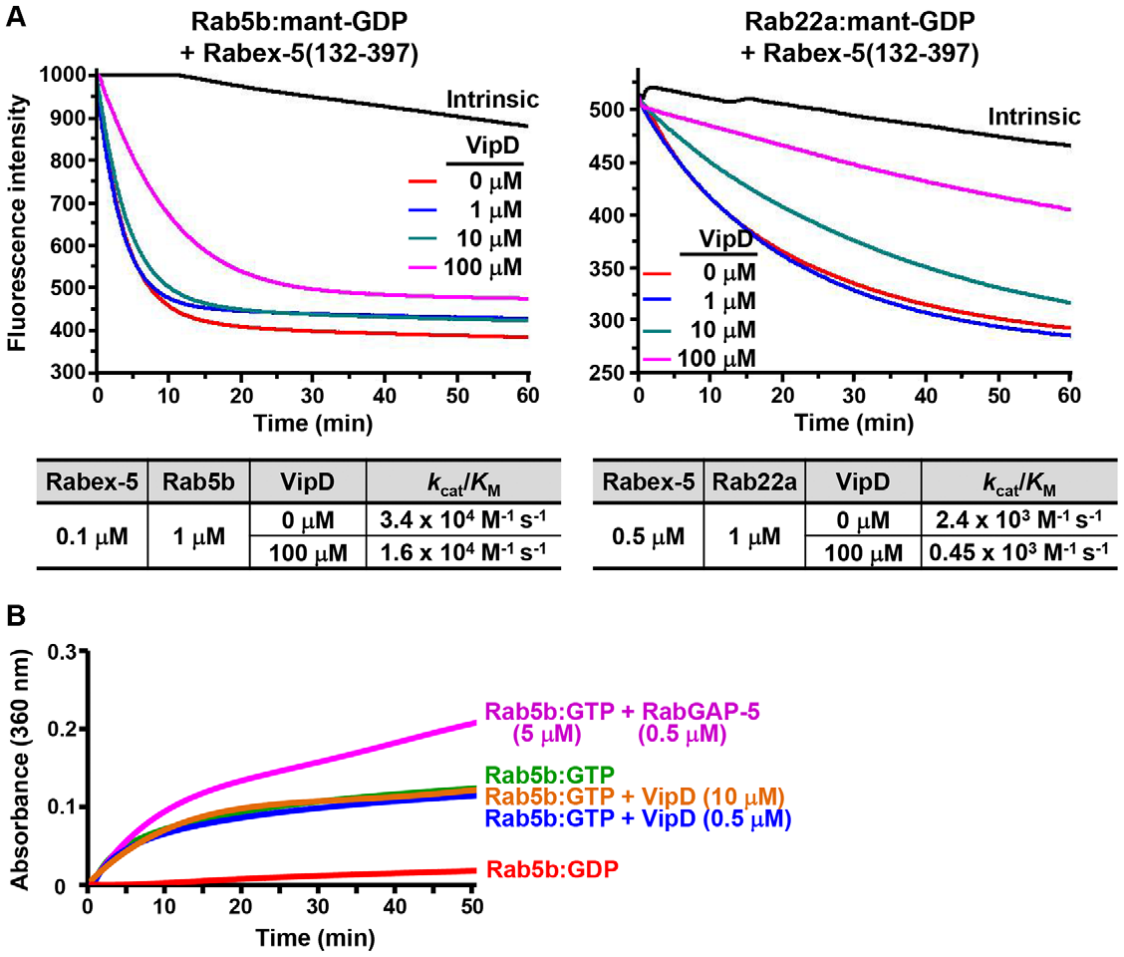
GEF and GAP activity assays. (A) VipD slightly affects the GEF activity of Rabex-5. In the presence of GTP (0.2 mM) and VipD at the indicated concentration, Rab5b(1-190):mant-GDP (1 µM) was reacted with Rabex-5(132-397) (0.1 µM). The same experiment was performed for Rab22a(1-175):mant-GDP (1 µM), but at an elevated concentration of Rabex-5(132-397) (0.5 µM). The decreased fluorescence as a result of mant-GDP-to-GTP exchange was continuously monitored and used to deduce the *k*_cat_/*K*_M_ values (M^−1^s^−1^). (B) VipD does not activate the GTPase activity of Rab5b. In the presence of 5 mM GTP, Rab5b(1-190):GTP (5 µM) was reacted with RabGAP-5(1-451) or VipD at the indicated concentration. Phosphate production in the reaction mixtures was measured using the EnzChek phosphate assay kit at 360 nm.

Given the protein-binding analyses and the minor structural similarity between the C-terminal domain of VipD and the Vps9 domain of Rabex-5 (Figure S1B), we suspected that VipD might have a GEF activity toward Rab5 and Rab22. This possibility was tested by fluorescence resonance energy transfer assay using 2′/3′-*O*-(*N*′-methylanthraniloyl)-GDP (mant-GDP)-loaded Rab5b(1-190) and Rab22a(1-175). VipD exhibited no observable GEF activity toward the two Rabs (Not shown; indirectly shown in Figure 4A), and thus it is not a GEF for the two Rabs. An alternative possibility that VipD might competitively inhibit a cellular GEF for Rab5 and Rab22 was examined by performing a GEF activity assay with Rab5b(1-190):mant-GDP, Rab22a(1-175):mant-GDP and the Vps9 domain (residues 132-397) of Rabex-5, which is a strong and a comparatively weak GEF for Rab5 and Rab22, respectively [28]. VipD noticeably but very weakly inhibited the GEF activity of the Vps9 domain; 1000 molar excess of VipD over the Vps9 domain decreased *k*_cat_/*K*_M_ by only about two folds for Rab5b(1-190) (Figure 4A; left panel). VipD inhibited the weak GEF activity of Rabex-5 toward Rab22a more evidently. However, also in this case, only five folds decrease of *k*_cat_/*K*_M_ was detected when VipD was present at 1000 molar excess over Rabex-5 (Figure 4A; right panel). These results indicate that VipD can interfere with the GEF activity of Rabex-5 but only slightly. These inhibitory effects presumably arise from the binding affinity of VipD for the GDP-bound forms of the two Rabs (Figure 3). Whether VipD could function as a GTPase-activating protein (GAP) for Rab5b was also tested by employing the GAP domain of RabGAP-5 (residues 1-451), a specific cellular GAP for Rab5 [37], and performing an enzyme assay designed to detect the phosphate ion released from GTP hydrolysis by Rab5b(1-190). Addition of the GAP domain markedly increased the GTP hydrolysis (Figure 4B). In contrast, VipD had no effect on the GTP hydrolysis (Figure 4B), demonstrating that VipD does not function as a GAP for Rab5b.

### VipD abrogates the binding of downstream effectors to activated Rab5b and Rab22a

**Figure 5:**
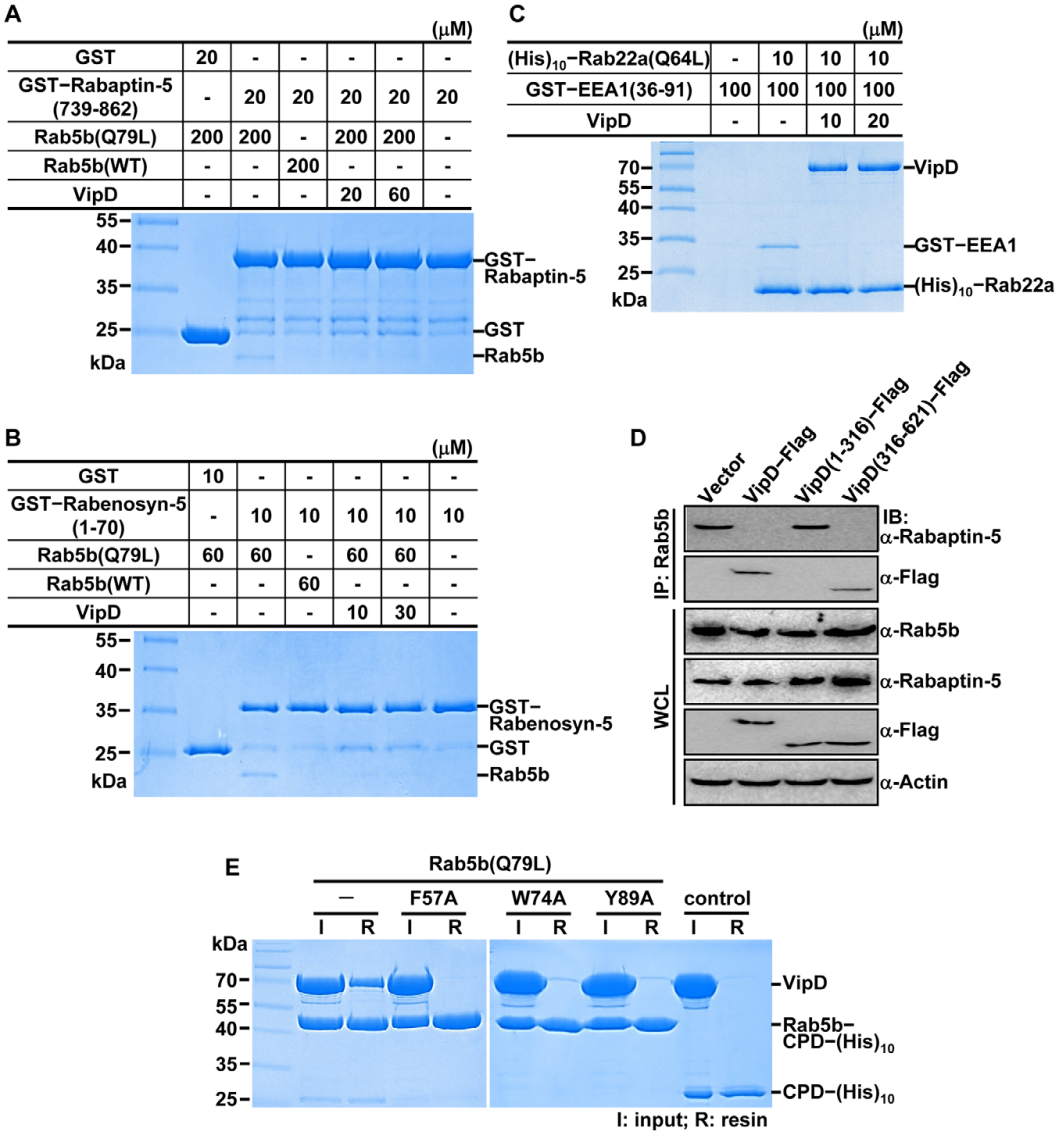
VipD disrupts the Rab:GTP–host effector interactions. (A–B) VipD disrupts the interaction between Rab5b:GTP and Rabaptin-5 or Rabenosyn-5. GST–Rabaptin-5(739-862) or GST–Rabenosyn-5(1-70) was bound to glutathione-agarose resin and incubated for 30 min with Rab5b(1-190;Q79L):GTP at a 1∶10 or 1∶6 molar ratio. The resin was washed to remove the unbound Rab5b protein and then VipD was added at the indicated concentration. After 30 min incubation, the resin was washed again and the resin-bound proteins were visualized on a denaturing gel. Rab5b was coprecipitated with Rabaptin-5 or Rabenosyn-5 in the absence of VipD (*A* and *B*; lane 3), but washed out in the presence of VipD (*A* and *B*; lanes 5 and 6). The effectors did not interact with GDP-bound Rab5b (*A* and *B*; lane 4). (C) VipD readily displaced EEA1 bound to Rab22a:GTP. (His)_10_-tagged Rab22a(1-175;Q64L):GTP was bound to Co^2+^ resin and incubated with GST-tagged EEA1(36-91) alone (lane 3) or together with VipD at the indicated concentration (lanes 4 and 5). The resin was washed after 30 min incubation and the resin-bound proteins were visualized on a denaturing gel. The interaction between GST–EEA1 and Rab22a (lane 3) was disrupted by VipD even at large molar excess of EEA1 over VipD (lanes 4 and 5). (D) Cell-based binding assay. The indicated Flag-tagged VipD proteins were stably expressed in RAW264.7 macrophages and the effect of VipD expression on the endogenous Rab5b–Rabaptin-5 interaction was assessed by immunoprecipitation and immunoblotting. Full-length VipD and VipD(316-621) displaced Rab5b from Rabaptin-5, while VipD(1-316) did not bind Rab5b and has no effect on the interaction between Rab5b and Rabaptin-5. (E) Three hydrophobic residues of Rab5b are critical for the interaction with VipD. A (His)_10_ pull-down assay was performed with VipD and the four indicated Rab5b variants containing a CPD–(His)_10_ tag. The binding of VipD to Rab5b(1-190;Q79L):GTP (lanes 2 and 3) was abrogated by the alanine substitution of Phe57, Trp74 and Tyr89 forming a conserved hydrophobic triad (lanes 4 to 9).

Another possibility was that VipD binding to activated Rab5b and Rab22a prevents the interactions with their direct downstream effectors. Rabaptin-5 and Rabenosyn-5 bind directly to activated Rab5 and mediate endocytic membrane docking and fusion as well as early endosomal trafficking [38]–[41]. In a glutathione S-transferase (GST) pull-down assay, GST-tagged Rabaptin-5(739-862), encompassing the Rab5-binding domain of the protein [40], bound to the GTP-bound form, but not to the GDP-bound form of Rab5b (Figure 5A; lanes 3 and 4). This complex was disrupted when VipD was challenged in a 1∶1 molar ratio with GST–Rabaptin-5(739-862) (Figure 5A; lane 5). Likewise, VipD disrupted the interaction between Rab5b(1-190;Q79L):GTP and GST-tagged Rabenosyn-5(1-70), which includes the Rab5-binding domain of the protein (Figure 5B). We also examined whether VipD affects the interaction between Rab22a and its effector protein early endosome autoantigen 1 (EEA1), whose N-terminal C_2_H_2_ Zn^2+^ finger domain is necessary for binding Rab22a and for controlling endosomal trafficking [42], [43]. VipD aptly displaced GST–EEA1(36-91) bound to Rab22a(1-175;Q64L):GTP even at a 1∶10 molar ratio between VipD and EEA1(36-91) (Figure 5C; lanes 3 to 5). Consistently with these *in vitro* displacement assays, the endogenous association between Rab5b and Rabaptin-5 in RAW264.7 macrophages was disrupted by the expression of full-length VipD or VipD(316-621), but not by the expression of VipD(1-316) (Figure 5D). What would be the basis for the observed competitive binding of VipD to the activated Rabs? The Rab effectors commonly make contacts with a predominantly nonpolar surface of their cognate Rab, on which three highly conserved apolar residues (Phe57, Trp74 and Tyr89 in human Rab5b; see Figure S8) form a hydrophobic triad that is critical for the binding interaction [38], [40], [42], [44], [45]. In a (His)_10_ pull-down assay, three Rab5b variants with an alanine substitution of one of the three residues exhibited no or barely detectable interaction with VipD (Figure 5E), pointing that VipD also recognizes the hydrophobic triad and therefore competes with the effector molecules for binding to the activated Rabs. Together, these results indicate that the C-terminal domain of VipD is able to counteract the downstream signaling from the activated form of Rab5 and Rab22.

### Blockade of endosomal trafficking by VipD

**Figure 6:**
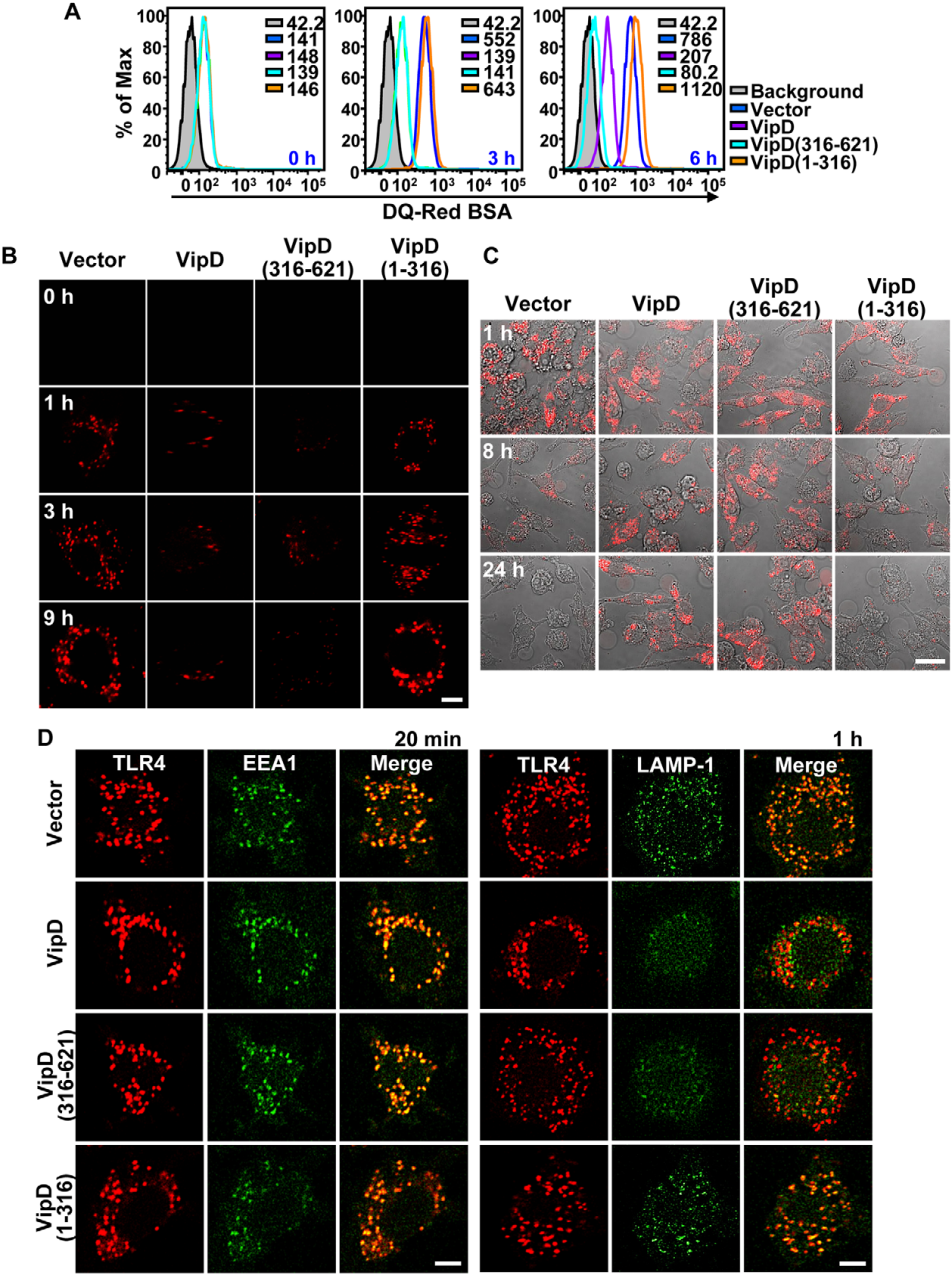
VipD blocks the endocytic degradation pathway in macrophages. (A–B) Time-course flow cytometric (*A*) and confocal microscopic (*B*) analyses of the lysosomal degradation of DQ-Red BSA in LPS-treated RAW264.7 macrophages stably expressing the indicated VipD proteins. The fluorescence emission was greatly suppressed in cells expressing full-length VipD or VipD(316-621). The scale bar indicates 5 µm. (C) Blockade of degradation of phagocytosed bacteria. RAW264.7 macrophages expressing the indicated VipD proteins were fed with mCherry-expressing *E. coli* (shown in red) and visualized by confocal microscopy at the indicated time points. Disappearance of the fluorescence indicates the digestion of internalized *E. coli*. The scale bar indicates 50 µm. (D) Tracking LPS-induced endocytosis of TLR4 in RAW264.7 macrophages. Cells expressing the indicated VipD proteins were fixed at 20 min (left) or 1 h (right) after LPS treatment. TLR4, EEA1 and LAMP-1 were immunostained and visualized. TLR4 internalization was clearly observable in LPS-treated cells but not in untreated cells (see Figure S9). The internalized TLR4 colocalized with EEA1, but not with LAMP-1 in cells expressing full-length VipD or VipD(316-621). The LAMP-1 signal was consistently weak in cells expressing full-length VipD or VipD(316-621) (column 5; rows 2 and 3). The reason is unclear, but it may be due to the blockade of endosomal maturation by the VipD proteins, which could cause attenuated LAMP-1 localization to late endosomes. The scale bars indicate 5 µm.

The capacity of VipD to disrupt the interactions between the three effectors and Rab5b or Rab22a strongly suggested that VipD interferes with endosomal trafficking leading to the degradation of endocytic materials. We therefore analyzed the effect of VipD expression on the transport and the degradation of exogenously added DQ-Red bovine serum albumin (BSA), which emits red fluorescence upon proteolytic degradation and is used as a sensitive indicator of lysosomal activity. In lipopolysaccharide (LPS)-treated RAW264.7 mouse macrophages, the degradation of DQ-Red BSA was significantly attenuated in cells stably expressing full-length VipD or VipD(316-621) compared with that in cells expressing vector alone or VipD(1-316) (Figures 6A and 6B). Furthermore, expression of full-length VipD or VipD(316-621) also blocked the degradation of phagocytosed *E. coli* in RAW264.7 cells, while the bacteria were disintegrated within 24 hours in macrophages expressing vector alone or VipD(1-316) (Figure 6C). Next, time-course confocal microscopy was performed to identify which step of the endocytic degradation pathway was affected by VipD (Figure 6D). LPS is recognized by the Toll-like receptor 4 (TLR4)–MD-2 complex and induces endocytic internalization and consequent lysosomal degradation of the receptor complex [46]. TLR4 was internalized into the RAW264.7 macrophage cytoplasm and colocalized with the early endosomal marker EEA1 within 20 min after LPS treatment, regardless of the expression of any VipD constructs (Figure 6D; left panels), indicating that VipD does not interfere with the formation of endocytic vesicles or their heterotypic fusion with early endosomes. Critically, in 1 hour after LPS treatment, TLR4 colocalized with the late endosomal/lysosomal marker lysosome-associated membrane protein-1 (LAMP-1) in cells expressing vector alone or VipD(1-316), but not in cells expressing full-length VipD or VipD(316-621) (Figure 6D; right panels). These results suggest that VipD might block the endosome maturation step in macrophage cells via the C-terminal domain.

Stably expressed Rab5c was shown to be excluded from *L. pneumophila*-containing phagosomes in HeLa cells [47]. We sought to examine whether endogenous Rab5 might be excluded, and if it is, VipD might be responsible for the exclusion. C57BL/6 mouse bone marrow-derived macrophages (BMDM) and three different *L. pneumophila* mutant strains were prepared: Lp03 (*dotA*-deficient type IV secretion system-defective), Δ*flaA* (flagellin-gene deficient) and Δ*vipD*/Δ*flaA* (*vipD* and *flaA*-deficient). However, we found that endogenous Rab5b does not localize to the LCV in *L. pneumophila*-infected macrophages regardless of the strain background (Figure S10A; columns 1–3). In a positive control experiment, endogenous Rab1b localized to the LCV in cells infected by the Δ*flaA* or Δ*vipD*/Δ*flaA* strain but not in cells infected by the Lp03 strain (Figure S10A; columns 4–6). Similar results were obtained with two different cell lines (macrophage-like human monocytic leukemia U937 and human alveolar basal epithelial A549 cells), which were infected by the *L. pneumophila* strain Lp02 (wild-type) or Δ*vipD* (*vipD*-deficient). In both type of cells, Rab5b did not localize to the LCV, irrespective of the presence of VipD (Figures S10B and S10C; rows 1–2). In contrast, Rab1b localized to the LCV in both types of cells infected by the Lp02 strain (Figures S10B and S10C; row 3). These observations reinforce the notion that Rab5 is excluded from the LCV, and suggest that at least VipD is not responsible for this exclusion. As expected, the Rab5 effectors EEA1 and Rabaptin-5 did not localize to the LCV, irrespective of the presence of VipD in these infected cells (Figures S10B and S10C; rows 4–7).

## Discussion

*L. pneumophila* resides and replicates in macrophages, which is at the forefront against infectious agents. To understand *L. pneumophila*'s strategies to evade the immune defense of macrophages, it is critical to know how pathogen's effector proteins manipulate host molecules. However, such information is yet very limited. Through elegant studies [6]–[12], [17], [48]–[51], a number of *L. pneumophila* effectors, SidM/DrrA, SidD, LepB, AnkX and LidA, have been identified to target host Rab proteins, especially and commonly Rab1, a key regulator of ER-to-Golgi vesicle trafficking. Dysregulation of Rab1 by these effectors enables *L. pneumophila* to divert ER-derived vesicles to the LCV for the supply of nutrients and membrane components, highlighting that ER-to-Golgi vesicle trafficking is an important target for the intracellular growth of the pathogen. The study presented herein shows that endosomal vesicle trafficking is also targeted by *L. pneumophila* via VipD that blocks downstream signaling from Rab5 and Rab22. These two Rabs compose a Rab22–Rabex-5–Rab5 signaling relay [52], where activated Rab22 recruits Rabex-5, the GEF promoting the GDP-to-GTP exchange on Rab5 [28], [29]. Activated Rab5 then recruits downstream effector proteins such as Rabaptin-5, Rabenosyn-5 and EEA1, which mediate diverse endosomal processes including vesicle fusion and membrane trafficking [39], [41], [53]. In addition, Rab22a regulates the formation of tubular recycling endosomes, which are necessary for endosome-to-plasma membrane recycling trafficking of internalized materials [31]. We show that VipD specifically and potently interacts with the two endosomal Rabs, blocking their binding interactions with the three downstream effectors through its C-terminal domain.

In the interaction of VipD with Rab5 and Rab22, three features are outstanding. First, VipD primarily targets the activated form of the two Rabs. Second, while activated Rab5 and Rab22 interact with their effector molecules weakly (*K*_D_>0.9 µM) [38], [42], the binding affinity of VipD for these Rabs is exceedingly higher (*K*_D_<254 nM). Third, VipD recognizes the conserved hydrophobic triad (Phe-Trp-Tyr), which is a common binding motif in diverse Rabs for the interaction with their downstream effector molecules [38], [40], [42], [44], [45]. These three features should enable VipD to potently block the downstream signaling from Rab5 and Rab22 by abrogating their association with the three effector molecules we tested in this study and probably with other effectors. To our knowledge, VipD is the first established example of a pathogen protein that antagonizes downstream signaling through binding to an activated Rab to competitively inhibit the binding of effector molecules. Of note, VipD does not interact with Rab7 (Figure S7), which replaces Rab5 on early endosomes [54] and mediates endosomal-lysosomal trafficking [55]. VipD also does not interact with Rab4b, Rab9a, Rab14 and Rab21 (Figure S7), which are known to mediate endosome-related trafficking [30]. Therefore, the observed endosomal trafficking block by VipD is most likely through selectively inhibiting the function of Rab5a, Rab5b, Rab5c and Rab22a.

In this study, we also confirmed that VipD has a phospholipase A_2_ activity and that Ser73 and Asp288, invariant in cPLA_2_, VipD, VpdA, VpdB and ExoU [22], constitute a catalytic dyad in VipD (Figures 1B and 1C). Since the N-terminal lipase domain of VipD is dispensable for VipD to localize to endosomes (Figures 2 and S3), to bind Rab proteins (Figure 3D) and to perturb endosomal trafficking (Figures 6 and S4B), the role of this domain is elusive. As VipD localizes to endosomes, one possibility is that VipD exhibits its catalytic activity on the endosomal membrane, the consequence of which remains to be elucidated.

In summary, the structural and biochemical analyses identified VipD as a signal blocker disabling the key endosomal regulators Rab5 and Rab22. As phagocytic vesicles could undergo fusion with lysosomes, our findings raise an important question of whether VipD facilitates the survival of *L. pneumophila* in macrophage, which needs further investigation. Our observations also form rational grounds for future investigations to delineate the role of the lipase activity of VipD and to decipher the functional roles of the C-terminal domain of the VipD-related bacterial effectors VpdA and VpdB, which are also translocated into host cells.

## Materials and Methods

### Crystallization and structure determination of VipD

The crystals of native VipD(1-575) were obtained by the hanging-drop vapor diffusion method at 22°C by mixing and equilibrating 1.5 µL of the final VipD(1-575) sample (16 mg/mL) and 1.5 µL of a precipitant solution containing 100 mM Tris-HCl (pH 8.0), 1.0 M ammonium citrate tribasic (pH 7.0) and 10 mM MgCl_2_. The crystals of selenomethionine-substituted VipD(1-575) grew from a mixture of 100 mM MES (pH 6.0) and 1.3 M ammonium sulfate. Before data collection, the crystals were immersed in the precipitant supplemented with 30% glycerol and incubated overnight at −20°C. This dehydration process at high glycerol concentration improved the resolution of X-ray diffraction; from typical 5 Å up to 2.9 Å. The crystals were plunged into liquid nitrogen before X-ray data collection. X-ray data sets were collected using synchrotron X-ray radiation. The structure was determined by single-wavelength anomalous dispersion phasing using a selenomethionine-substituted VipD(1-575) crystal with the programs SHELX [56] and autoSHARP [57]. Subsequently, model building and refinement were carried out using the programs COOT [58] and CNS [59]. The final model does not include residues 559–575, whose electron densities were not observed or very weak. Crystallographic data statistics are summarized in Table 1.

### Preparation of proteins for crystallization and *in vitro* assay

Full-length VipD(wild-type, S73A or D288A), VipD(1-575), VipD(316-621), 31 different Rab constructs, the GEF domain of Rabex-5, the Rab5-binding domains of Rabaptin-5 and Rabenosyn-5, the Rab22-binding domain of EEA1, and the GAP domain of RabGAP-5 were prepared for crystallization or biochemical assays, the details of which are described in Text S1.

### Protein binding analysis

For (His)_10_ pull-down assays, 25 µM of Rab–CPD–(His)_10_ and 37.5 µM of VipD or VipD(316-621) were incubated at room temperature for 30 min and mixed with 30 µL of Co^2+^ resin. The resin was washed four times with a buffer solution containing 20 mM Tris-HCl (pH 7.5), 100 mM NaCl and 2 mM MgCl_2_, and subjected to denaturing polyacrylamide gel electrophoresis. For quantification of protein-protein interaction, ITC measurements were carried out at 25°C on a microcalorimetry system iTC200 (GE Healthcare). Protein samples were prepared in a buffer solution containing 20 mM Tris-HCl (pH 7.5) and 100 mM NaCl. The samples were centrifuged to remove any residuals prior to the measurements. Dilution enthalpies were determined in separate experiments (titrant into buffer) and subtracted from the enthalpies of the binding between the proteins. Data were analyzed using the Origin software (OriginLab).

### Cell-based assay

For the subcellular localization analysis, HeLa cells and mouse macrophage RAW264.7 cells were transfected with the pEYFP-N1 or pECFP-C1 vectors (Clontech) encoding Rab or VipD proteins and visualized by confocal microscopy. For the analysis of endocytic trafficking, RAW264.7 cells were transfected with the pCDH-CMV vector (System Biosciences) encoding VipD proteins, and stable cell lines were established by puromycin selection. The details of mammalian cell culture, immunoblotting, flow cytometry and live cell imaging are described in Text S1.

### Accession codes

The coordinates of the VipD(1-575) structure together with the structure factors have been deposited in the Protein Data Bank with the accession code 4AKF.

## Supporting Information

Figure S1 **Structural superposition.** (A) The N-terminal domain of VipD (cyan) is superposed on patatin (yellow; left) and cPLA_2_ (orange; right). (B) The C-terminal domain of VipD (magenta) is superposed on the Vps9 domain of Rabex-5 (green). The orientation of the VipD domains is the same as the top panel in Figure 1A. Only the secondary structures of VipD overlapping with those of the counterparts are labeled for clarity.
(TIF)

Figure S2 **Catalytic groove of VipD.** VipD(1-316) is presented as a transparent surface with the sticks for the catalytic dyad (Ser73 and Asp288) and the oxyanion hole residues (Gly42-Gly-Gly-Ala-Lys46). The β10-α14 loop covering the catalytic groove is shown in a ribbon drawing together with the flanking β10 and α14.
(TIF)

Figure S3 **VipD colocalizes with Rab5b and Rab22a in macrophages.** Shown are the confocal images of RAW264.7 macrophages transiently expressing YFP-tagged VipD proteins and CFP-tagged Rab5b(Q79L) or Rab22a(Q64L). VipD colocalized with Rab5b(Q64L) or Rab22a(Q64L). The scale bar indicates 10 µm.
(TIF)

Figure S4 **VipD interferes with the formation of tubular structures induced by Rab22a.** (A) Images of HeLa cells transiently expressing each of the three indicated forms of Rab22a. Rab22a(S19N) is a dominant negative form which is defective in binding GTP. The tubular structure was clearly observed with the Rab22a(Q64L) expression. (B) The formation of tubular structures disappeared by the coexpression of full-length VipD or VipD(316-621), but not by the coexpression of VipD(1-316). The middle panels are adapted from Figure 2B for comparison. The scale bars indicate 10 µm.
(TIF)

Figure S5 **Confocal images of HeLa cells expressing VipD and Rab proteins.** YFP-tagged VipD and CFP- or mCherry-tagged Rab proteins were transiently expressed individually or together in HeLa cells. The cells were visualized by confocal microscopy. The scale bars indicate 10 µm. (A) VipD colocalized with wild-type Rab5b and Rab22a. (B) VipD and Rab5b(Q79L) did not colocalize with Lysotracker Red, which was treated 30 min before visualization. (C) VipD colocalized with Rab5a(Q79L) and Rab5c(Q80L). (D) VipD did not colocalize with Rab22b(Q64L) (top). VipD and Rab22a(Q64L) did not colocalize with Rab22b(Q64L), either, when the three proteins were coexpressed together (bottom).
(TIF)

Figure S6 **RNA interference assay.** HeLa cells transiently expressing YFP-tagged VipD or CFP-tagged Rab proteins were treated with the indicated siRNAs and visualized by confocal microscopy. The treatment of siRNA blocked the expression of the target Rab proteins (first and second rows). The endosomal localization of VipD was not affected by the siRNA treatment (third row). The scale bar indicates 10 µm.
(TIF)

Figure S7 **(His)_10_ pull-down assay.** Full-length VipD and each of the indicated GDP-bound (top) or GTP-bound (middle) Rabs fused to CPD–(His)_10_ were incubated together with Co^2+^ resin, and a (His)_10_ pull-down assay was performed as in Figure 3. None of the Rabs exhibited a notable coprecipitation with VipD except Rab22a used as a control. The table lists the Rab proteins tested in the pull-down assay and the intracellular trafficking they are involved in.
(TIF)

Figure S8 **Sequence alignment of Rabs.** The twelve different Rab proteins presented in this manuscript are aligned. The highly conserved three nonpolar residues commonly involved in binding to host effectors and to VipD are indicated by asterisks. Conserved residues are highlighted by red (>90% similarity) or yellow (>60% similarity) columns.
(TIF)

Figure S9 **Tracking TLR4 in LPS-untreated RAW264.7 cells.** RAW264.7 cells expressing the indicated VipD proteins were fixed synchronously with the LPS-treated cells (see Figure 6D), immunostained and visualized. TLR4 remained mostly at the plasma membrane. The bottom right panel shows that the expression levels of the VipD proteins were similar. The scale bars indicate 5 µm.
(TIF)

Figure S10 **Macrophage infection assay.** (A–C) BMDM (*A*), U937 (*B*) and A549 (*C*) cells were infected with the indicated *L. pneumophila* strains at the multiplicities of infection of 25 (*A*; columns 1–3), 10 (*A*; columns 4–6), 3 (*B*) and 10 (*C*). At 15 min (*A*; columns 1–3), 60 min (*A*; columns 4–6), 30 min (*B*) and 90 min (*C*) post infection, respectively, cells were visualized by confocal microscopy after staining with DAPI and antibodies against Rab5b, Rab1b, EEA1, Rabaptin-5 and *L. pneumophila* (denoted as *Lp* in *B* and *C*). The flagellin-encoding *flaA* gene was deleted to suppress rapid cell death of BMDM cells. The rates of Rab1b-positive LCVs were 0% (Lp03), 62.7% (Δ*flaA*) and 57.3% (Δ*vipD*/Δ*flaA*) (*A*, column 6). The quantification was based on 25 LCVs which were counted in each of three repeated experiments. The scale bars indicate 5 µm. (D) Expression of VipD in the cultured *L. pneumophila* strains (∼9×10^7^ cells) was checked by immunoblotting with the anti-VipD antibody. VpdB was detected as a control.
(TIF)

Text S1 **Details of protein preparation, biochemical assays and cellular assays.**
(DOC)
