# Supplementary figures and images for: VipD of Legionella pneumophila Targets Activated Rab5 and Rab22 to Interfere with Endosomal Trafficking in Macrophages
Source: PLoS Pathog. 2012 Dec 13;8(12):e1003082. doi: 10.1371/journal.ppat.1003082 (PMC3521694; doi:10.1371/journal.ppat.1003082)

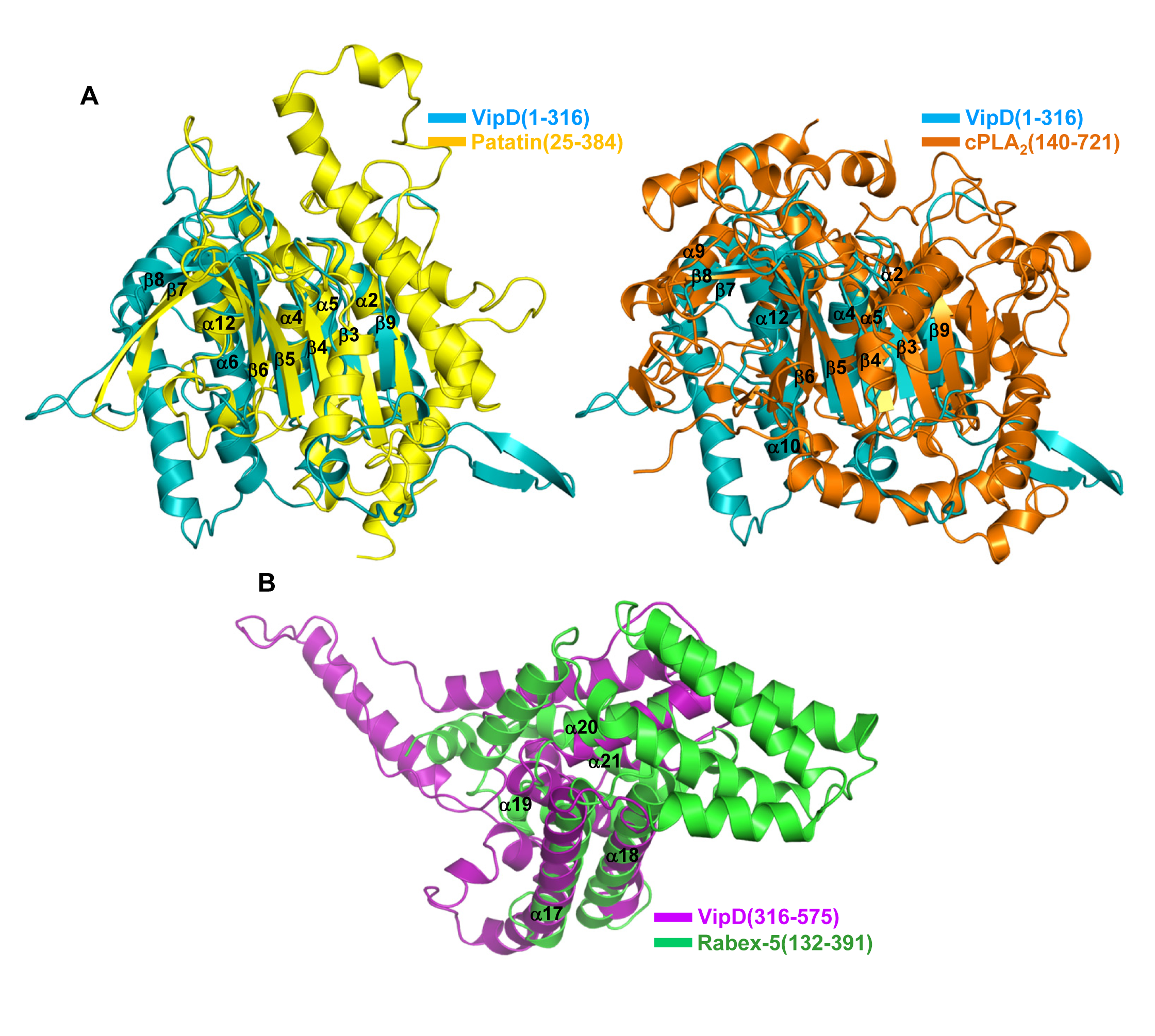

Supplement: Figure S1 — Structural superposition. (A) The N-terminal domain of VipD (cyan) is superposed on patatin (yellow; left) and cPLA2 (orange; right). (B) The C-terminal domain of VipD (magenta) is superposed on the Vps9 domain of Rabex-5 (green). The orientation of the VipD domains is the same as the top panel in Figure 1A. Only the secondary structures of VipD overlapping with those of the counterparts are labeled for clarity. (TIF) [file ppat.1003082.s001.tif]

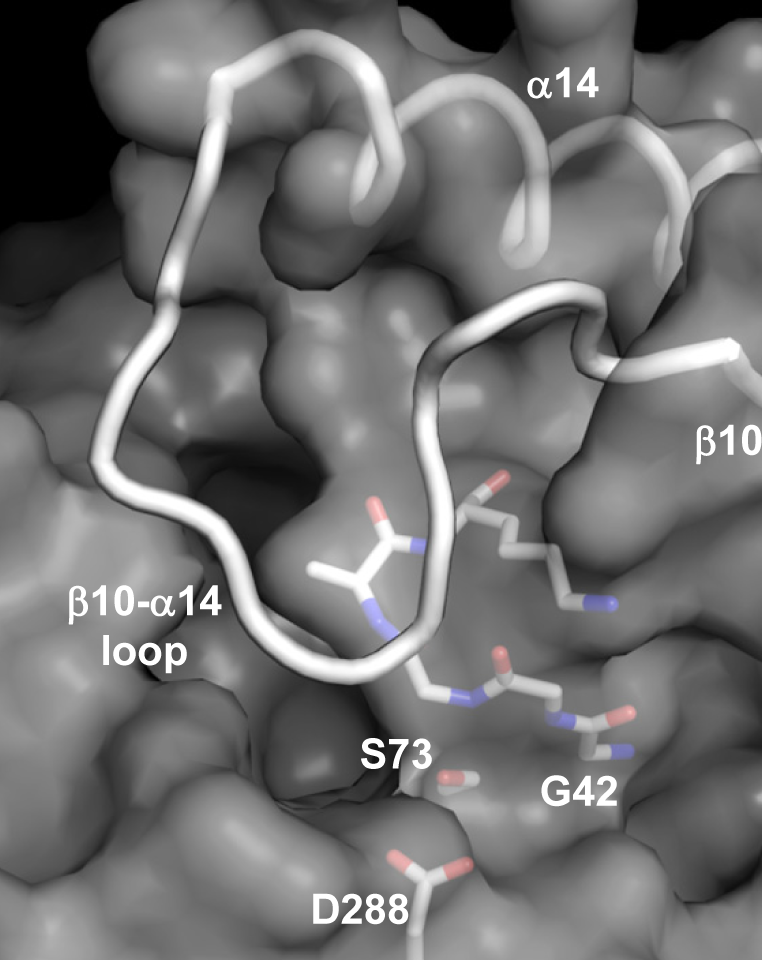

Supplement: Figure S2 — Catalytic groove of VipD. VipD(1-316) is presented as a transparent surface with the sticks for the catalytic dyad (Ser73 and Asp288) and the oxyanion hole residues (Gly42-Gly-Gly-Ala-Lys46). The β10-α14 loop covering the catalytic groove is shown in a ribbon drawing together with the flanking β10 and α14. (TIF) [file ppat.1003082.s002.tif]

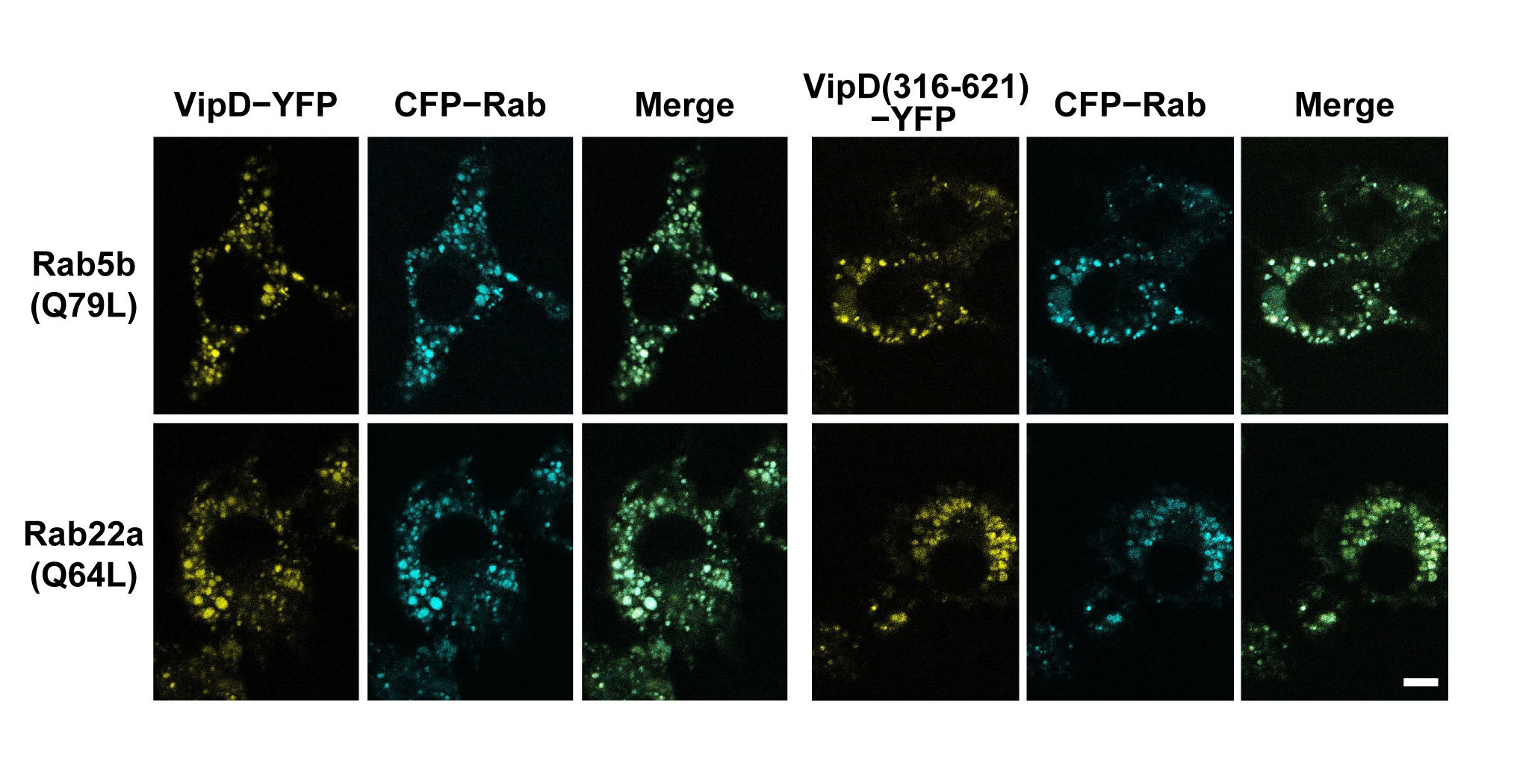

Supplement: Figure S3 — VipD colocalizes with Rab5b and Rab22a in macrophages. Shown are the confocal images of RAW264.7 macrophages transiently expressing YFP-tagged VipD proteins and CFP-tagged Rab5b(Q79L) or Rab22a(Q64L). VipD colocalized with Rab5b(Q64L) or Rab22a(Q64L). The scale bar indicates 10 µm. (TIF) [file ppat.1003082.s003.tif]

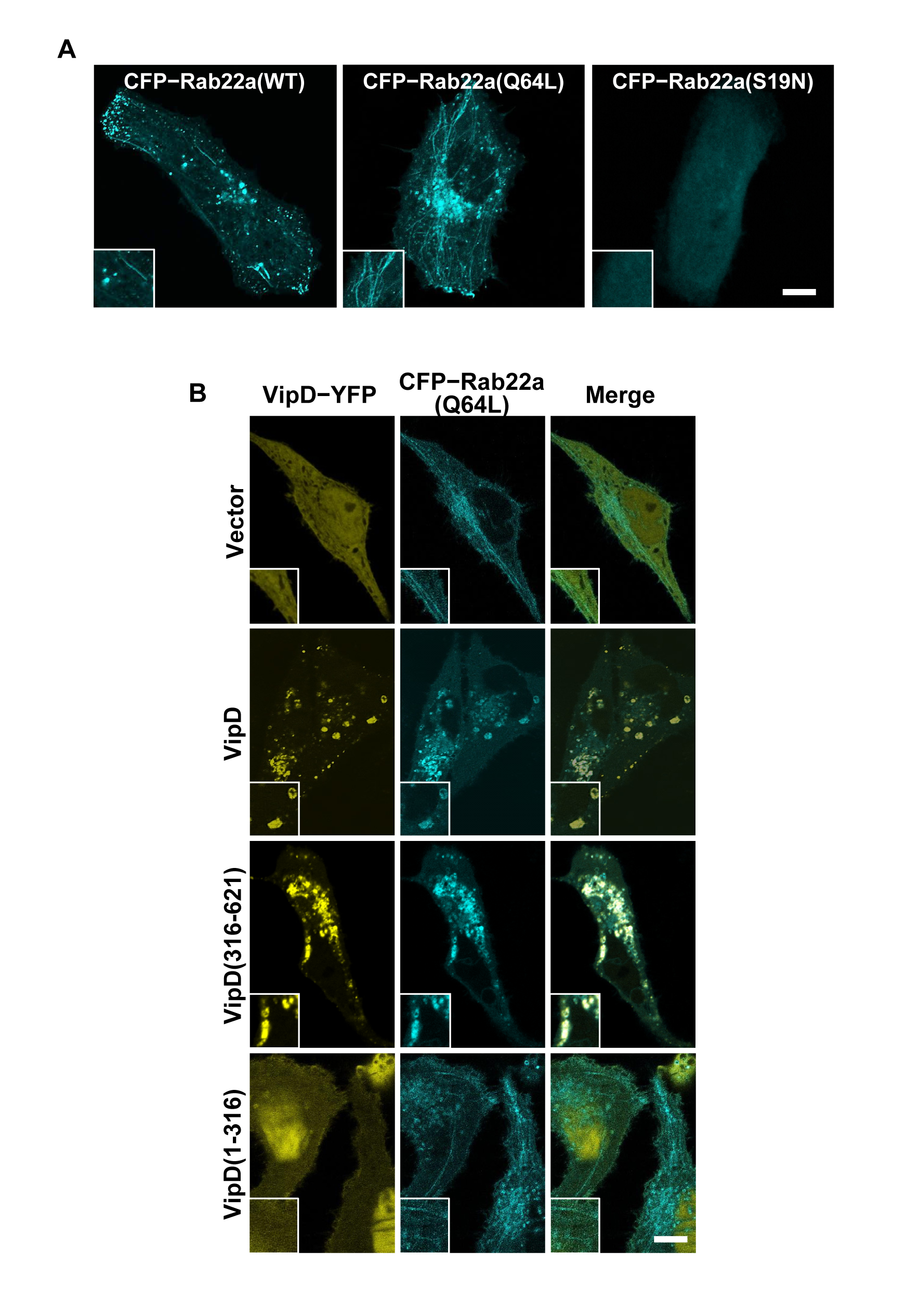

Supplement: Figure S4 — VipD interferes with the formation of tubular structures induced by Rab22a. (A) Images of HeLa cells transiently expressing each of the three indicated forms of Rab22a. Rab22a(S19N) is a dominant negative form which is defective in binding GTP. The tubular structure was clearly observed with the Rab22a(Q64L) expression. (B) The formation of tubular structures disappeared by the coexpression of full-length VipD or VipD(316-621), but not by the coexpression of VipD(1-316). The middle panels are adapted from Figure 2B for comparison. The scale bars indicate 10 µm. (TIF) [file ppat.1003082.s004.tif]

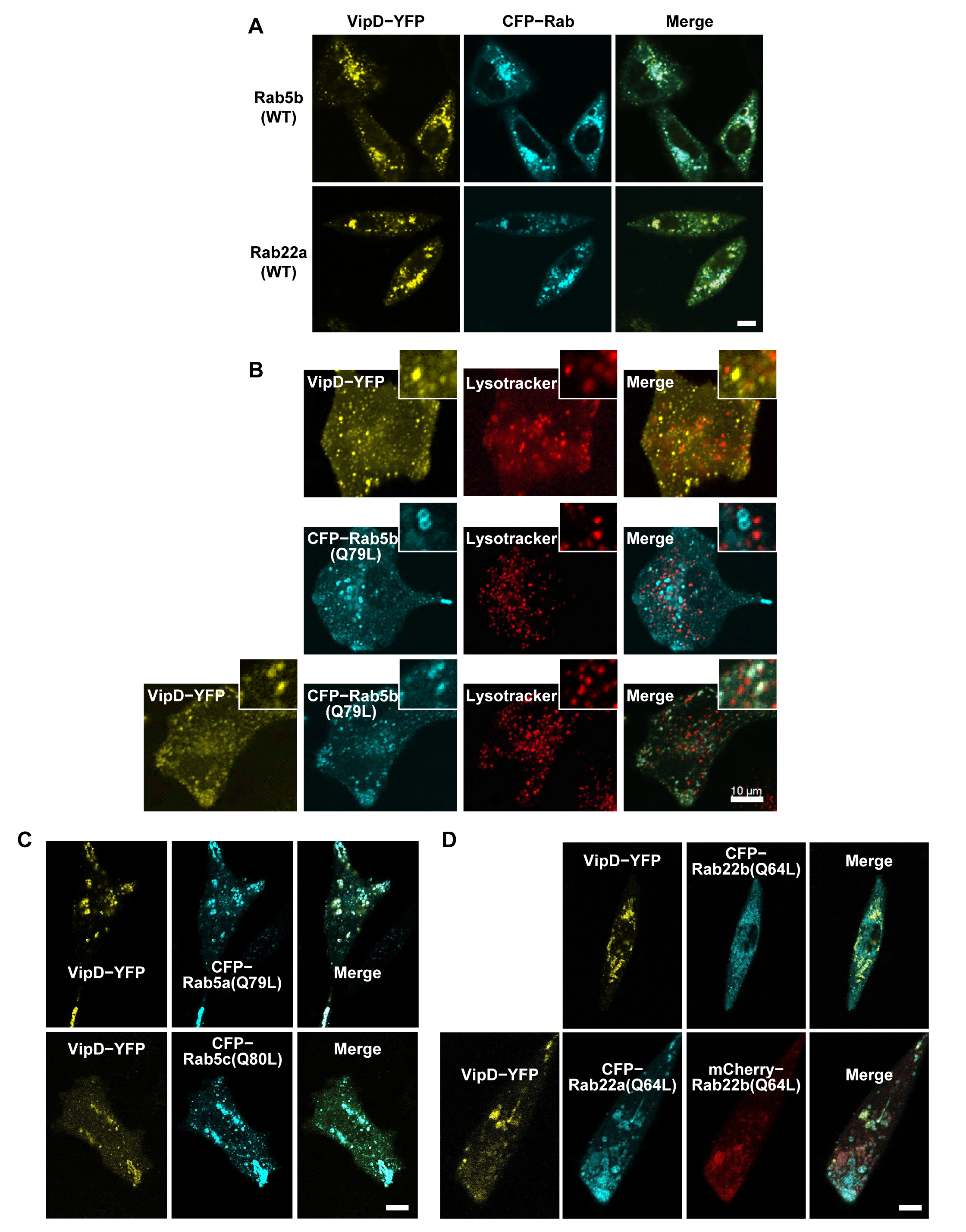

Supplement: Figure S5 — Confocal images of HeLa cells expressing VipD and Rab proteins. YFP-tagged VipD and CFP- or mCherry-tagged Rab proteins were transiently expressed individually or together in HeLa cells. The cells were visualized by confocal microscopy. The scale bars indicate 10 µm. (A) VipD colocalized with wild-type Rab5b and Rab22a. (B) VipD and Rab5b(Q79L) did not colocalize with Lysotracker Red, which was treated 30 min before visualization. (C) VipD colocalized with Rab5a(Q79L) and Rab5c(Q80L). (D) VipD did not colocalize with Rab22b(Q64L) (top). VipD and Rab22a(Q64L) did not colocalize with Rab22b(Q64L), either, when the three proteins were coexpressed together (bottom). (TIF) [file ppat.1003082.s005.tif]

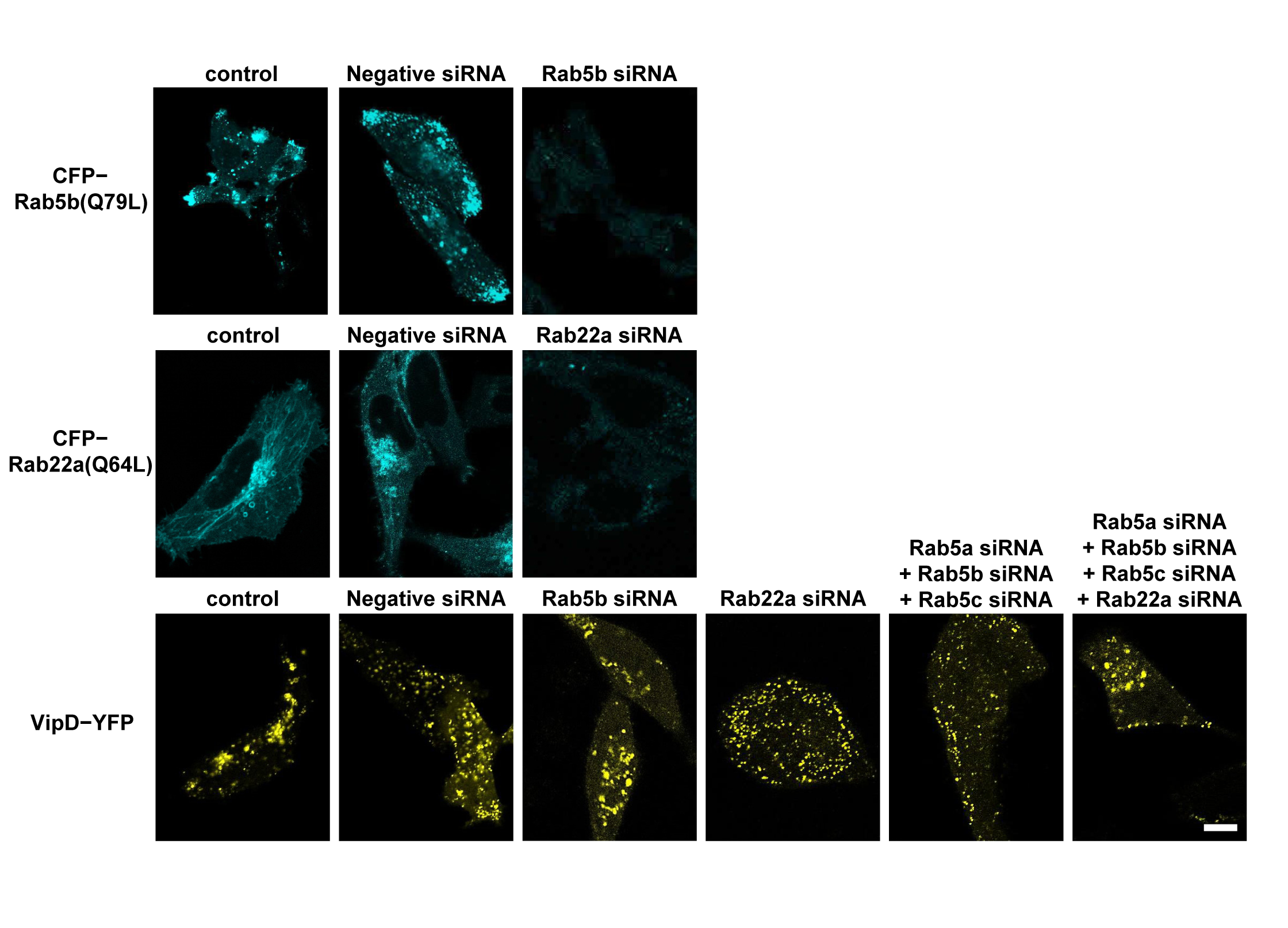

Supplement: Figure S6 — RNA interference assay. HeLa cells transiently expressing YFP-tagged VipD or CFP-tagged Rab proteins were treated with the indicated siRNAs and visualized by confocal microscopy. The treatment of siRNA blocked the expression of the target Rab proteins (first and second rows). The endosomal localization of VipD was not affected by the siRNA treatment (third row). The scale bar indicates 10 µm. (TIF) [file ppat.1003082.s006.tif]

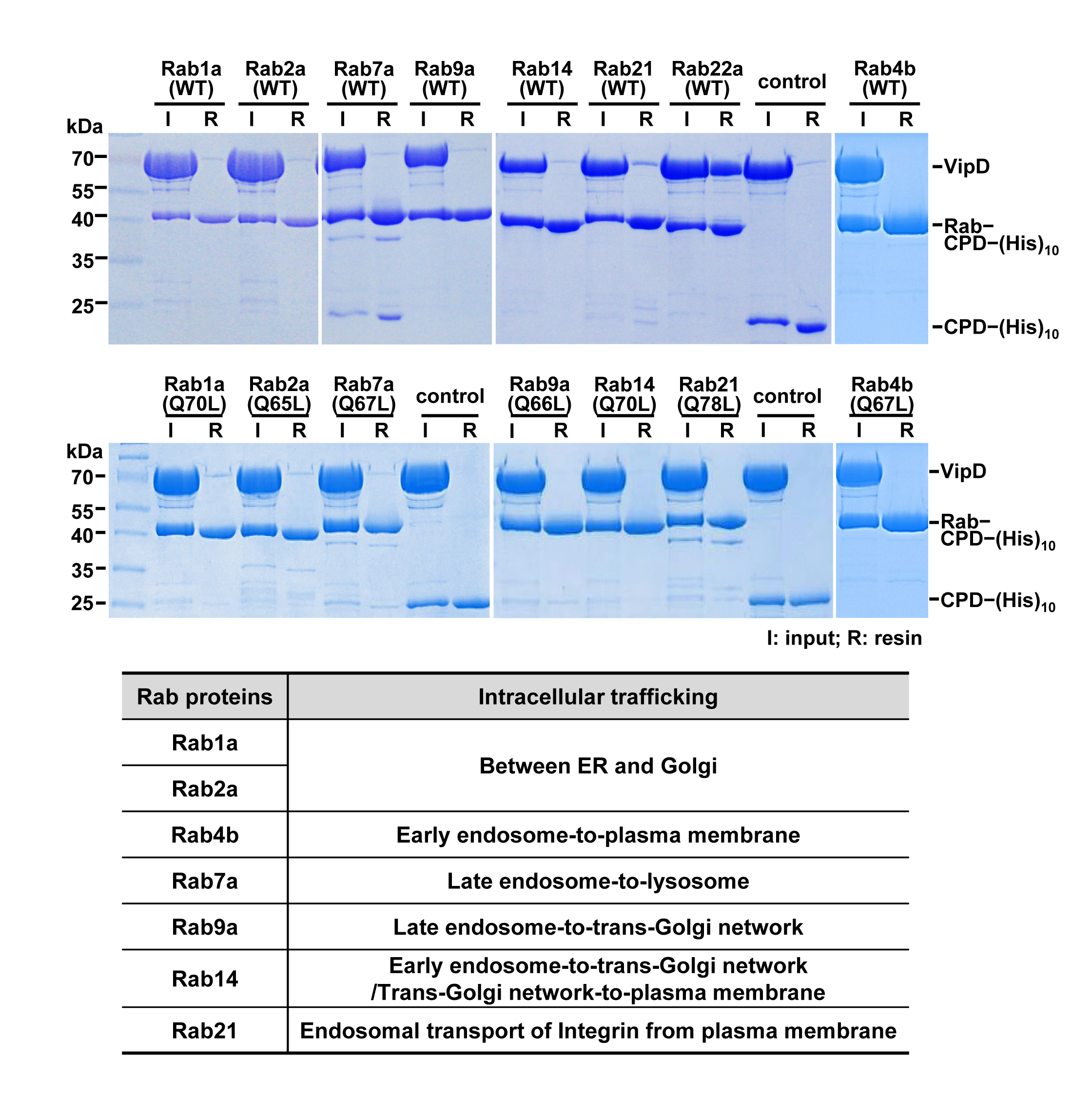

Supplement: Figure S7 — (His)10 pull-down assay. Full-length VipD and each of the indicated GDP-bound (top) or GTP-bound (middle) Rabs fused to CPD–(His)10 were incubated together with Co2+ resin, and a (His)10 pull-down assay was performed as in Figure 3. None of the Rabs exhibited a notable coprecipitation with VipD except Rab22a used as a control. The table lists the Rab proteins tested in the pull-down assay and the intracellular trafficking they are involved in. (TIF) [file ppat.1003082.s007.tif]

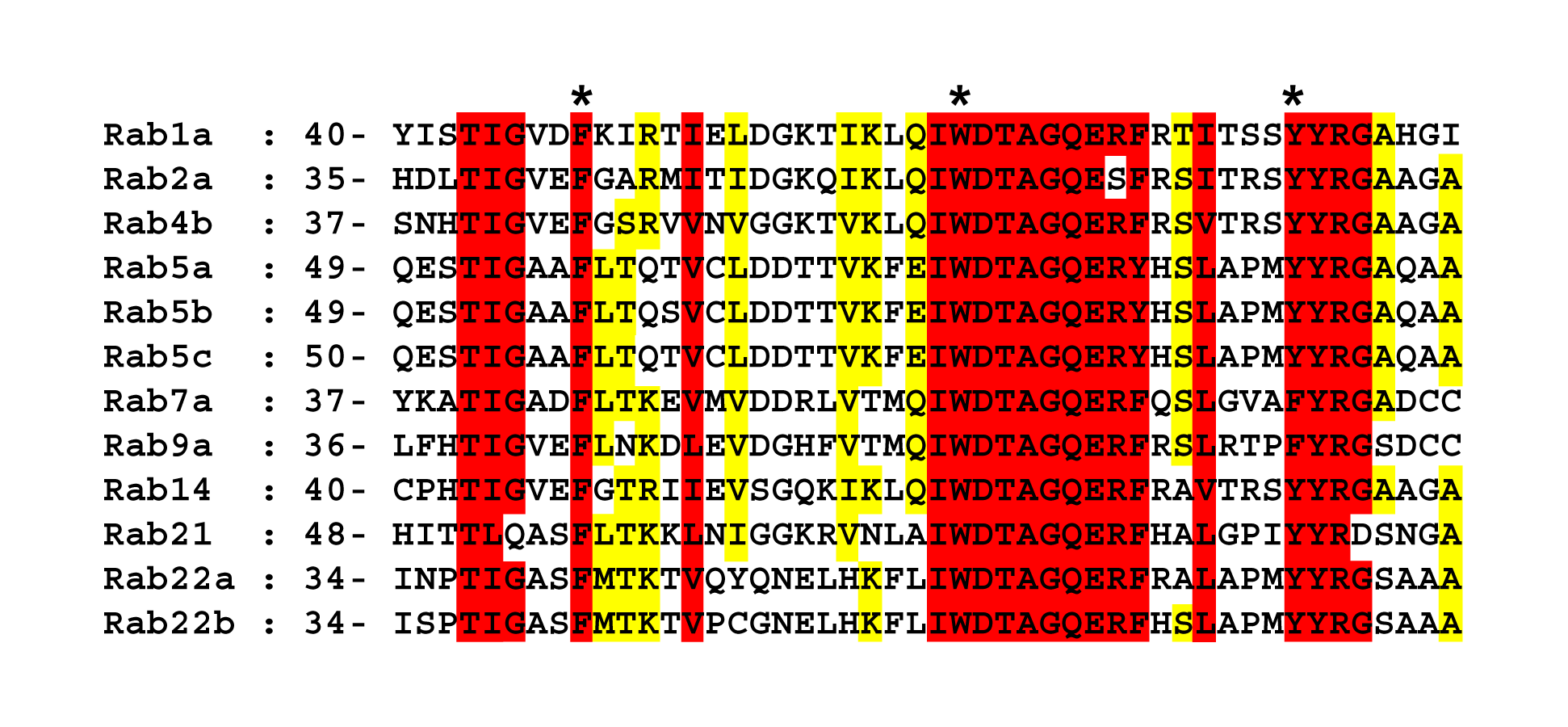

Supplement: Figure S8 — Sequence alignment of Rabs. The twelve different Rab proteins presented in this manuscript are aligned. The highly conserved three nonpolar residues commonly involved in binding to host effectors and to VipD are indicated by asterisks. Conserved residues are highlighted by red (>90% similarity) or yellow (>60% similarity) columns. (TIF) [file ppat.1003082.s008.tif]

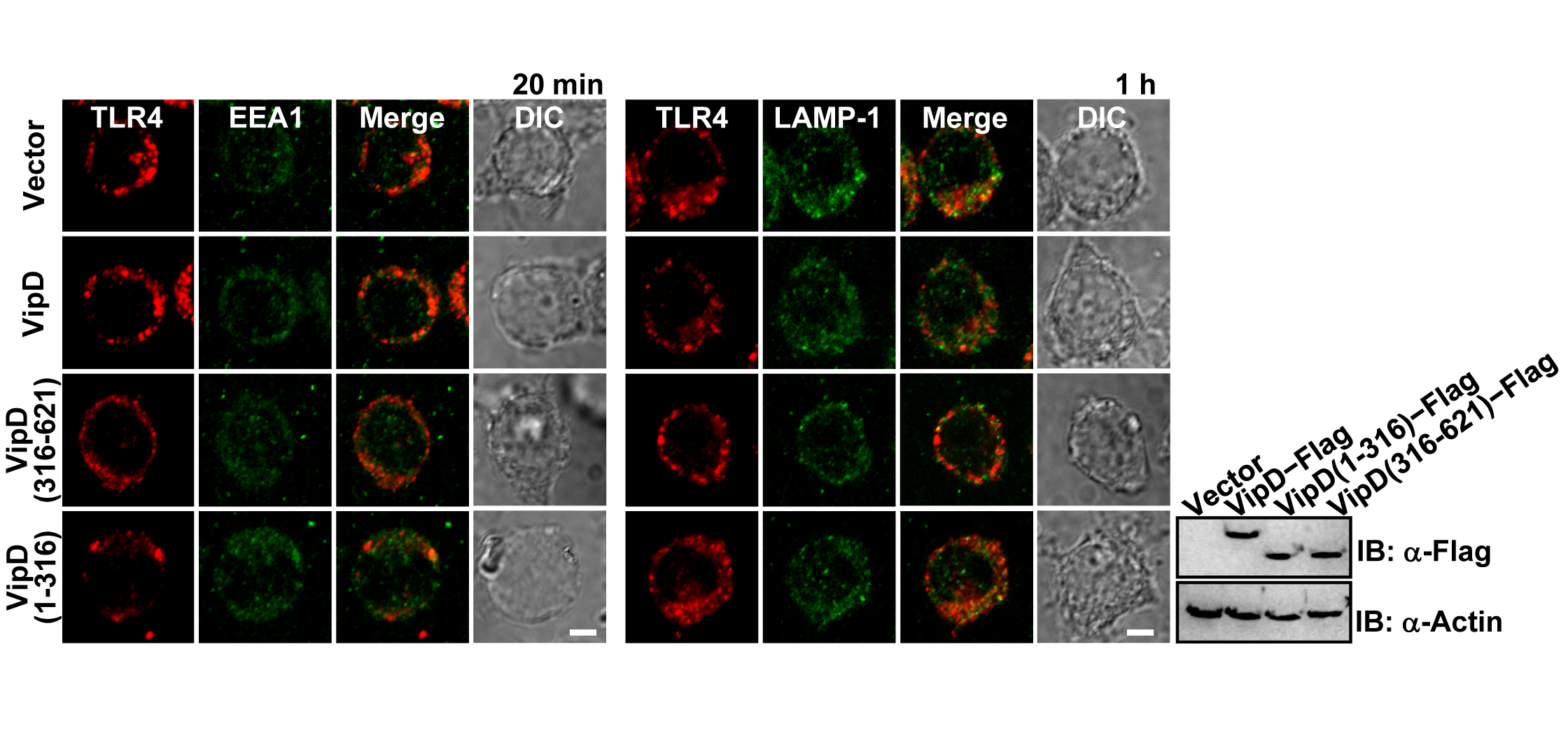

Supplement: Figure S9 — Tracking TLR4 in LPS-untreated RAW264.7 cells. RAW264.7 cells expressing the indicated VipD proteins were fixed synchronously with the LPS-treated cells (see Figure 6D), immunostained and visualized. TLR4 remained mostly at the plasma membrane. The bottom right panel shows that the expression levels of the VipD proteins were similar. The scale bars indicate 5 µm. (TIF) [file ppat.1003082.s009.tif]

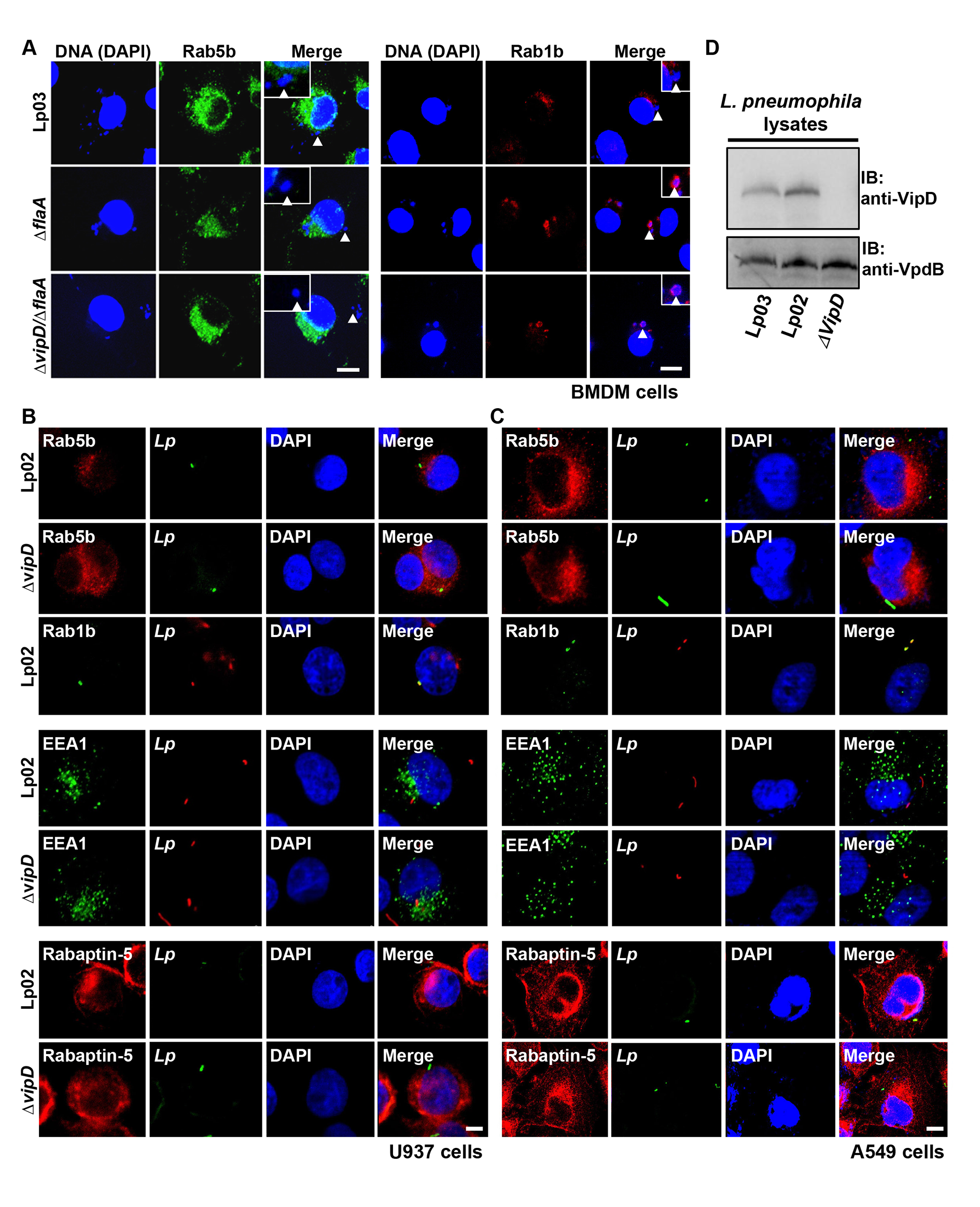

Supplement: Figure S10 — Macrophage infection assay. (A–C) BMDM (A), U937 (B) and A549 (C) cells were infected with the indicated L. pneumophila strains at the multiplicities of infection of 25 (A; columns 1–3), 10 (A; columns 4–6), 3 (B) and 10 (C). At 15 min (A; columns 1–3), 60 min (A; columns 4–6), 30 min (B) and 90 min (C) post infection, respectively, cells were visualized by confocal microscopy after staining with DAPI and antibodies against Rab5b, Rab1b, EEA1, Rabaptin-5 and L. pneumophila (denoted as Lp in B and C). The flagellin-encoding flaA gene was deleted to suppress rapid cell death of BMDM cells. The rates of Rab1b-positive LCVs were 0% (Lp03), 62.7% (ΔflaA) and 57.3% (ΔvipD/ΔflaA) (A, column 6). The quantification was based on 25 LCVs which were counted in each of three repeated experiments. The scale bars indicate 5 µm. (D) Expression of VipD in the cultured L. pneumophila strains (∼9×10^7^ cells) was checked by immunoblotting with the anti-VipD antibody. VpdB was detected as a control. (TIF) [file ppat.1003082.s010.tif]
