# Supplementary material for: VipD of Legionella pneumophila Targets Activated Rab5 and Rab22 to Interfere with Endosomal Trafficking in Macrophages
Source: PLoS Pathog. 2012 Dec 13;8(12):e1003082. doi: 10.1371/journal.ppat.1003082 (PMC3521694; doi:10.1371/journal.ppat.1003082)
Supplement: Text S1 — Details of protein preparation, biochemical assays and cellular assays. (DOC) [file ppat.1003082.s011.doc]

**Supplementary Methods**

**Preparation of VipD for crystallization**

Genomic DNA of *L. pneumophila* (ATCC 33152D-5) was the source for polymerase chain reaction to amplify the VipD gene. The DNA fragment encoding residues 1-575 out of a total of 621 residues of *L. pneumophila* VipD (entry: YP_096826.1) was cloned into the pGEX-4T-3 vector (GE Healthcare). The protein, referred to as VipD(1-575), was expressed in the *E. coli* BL21(DE3) RIG strain (Novagen) at 18 ^o^C overnight. Cell lysate was prepared with a lysis buffer containing 20 mM Tris (pH 7.5), 100 mM NaCl and 1 mM dithiothreitol and applied to glutathione-agarose resin (Novagen). GST−VipD(1-575) was eluted with the 10 mM reduced glutathione-containing lysis buffer and reacted with TEV protease to remove the N-terminal GST-tag. The protein was further purified using a HiTrap Q anion exchange column (GE Healthcare) and a HiLoad 26/60 Superdex 75 gel filtration column (GE Healthcare), equilibrated with the lysis buffer. Selenomethionine-substituted VipD was produced using the *E. coli* B834 (DE3) methionine auxotroph (Novagen) and purified as described above.

**Production of Rab proteins**

For (His)_10_ pull-down assays, the DNA fragments encoding the GTPase domain of 12 different Rabs, Rab1a(1-182), Rab2a(1-182), Rab4b(1-178), Rab5a(1-190), Rab5b(1-190), Rab5c(1-191), Rab7a(1-190), Rab9a(1-185), Rab14(1-189), Rab21(15-200), Rab22a(1-175) and Rab22b(1-175), were ligated into a vector derived from pET22b or pET30a (Novagen). The vectors were designed to produce a protein C-terminally fused to the cysteine protease domain of the *Vibrio cholera* MARTX toxin protein [1] with a (His)_10_-tag. Using the same vectors, the GTPase-defective mutant forms, Rab1a(1-182;Q70L), Rab2a(1-182;Q65L), Rab4b(1-178;Q67L), Rab5a(1-190;Q79L), Rab5b(1-190;Q79L), Rab5b(1-190;F57A and Q79L), Rab5b(1-190;W74A and Q79L), Rab5b(1-190;Q79L and Y89A), Rab5c(1-191;Q80L), Rab7a(1-190;Q67L), Rab9a(1-185;Q66L), Rab14(1-189;Q70L), Rab21(15-200;Q78L), Rab22a(1-175;Q64L) and Rab22b(1-175;Q64L), were also cloned. The fusion proteins were purified using HisPur Cobalt resin (Thermo Scientific) and a HiTrap Q anion exchange column. For ITC measurements and biochemical experiments, the DNA fragments coding for Rab5b(1-190;WT or Q79L) and Rab22a(1-175; WT or Q64L) were cloned into the 10H-GST-pPROEX HTa vector and the 10H-pPROEX HTa vector, respectively, which were derived from the pPROEX HTa vector (Invitrogen) to produce a protein with the N-terminal (His)_10_−GST-tag or (His)_10_-tag. These tags were cleaved with TEV protease after the glutathione-agarose or HisPur Cobalt column purification step and removed by a HiTrap Q anion exchange column.

**Production of VipD, Rabaptin-5, Rabenosyn-5, EEA1, Rabex-5 and RabGAP-5**

Each of the DNA fragments encoding the full-form (wild-type, S73A or D288A) or the C-terminal domain (residues 316-621) of VipD, the Rab5-binding domain of mouse Rabaptin-5 (residues 739-862) and human Rabenosyn-5 (residues 1-70) or the Rab22-binding domain of mouse EEA1 (residues 36-91) was cloned into the pGEX-4T-3 vector. The DNA fragments encoding the GEF domain of human Rabex-5 (residues 132-397) and the GAP domain of human RabGAP-5 (residues 1-451) were cloned into the 10H-pPROEx HTa vector and the pMAL-c2X vector (New England Biolabs), respectively. These proteins were expressed in the *E. coli* BL21(DE3) RIPL strain (Novagen) at 18 ^o^C overnight and purified similarly as the Rab proteins.

**Phospholipase A_2_ activity assay**

Phospholipase activity of VipD was measured according to the protocol provided with the EnzChek PLA_2_ assay kit (Molecular Probes). The red/green BODIPY PC-A2 (1-*O*-(6-BODIPY (dipyrromethene boron difluoride) 558/568-aminohexyl)-2-BODIPY FL C_5_-*sn*-glycero-3-phosphocholine) substrate was mixed with liposomes containing dioleoylphosphatidylcholine and dioleoylphosphatidylglycerol. The substrate-liposome mix was incubated with bee venom phospholipase A_2_ (5 units/mL) or VipD proteins (10 μM) on 96-well microplate at room temperature for 10 min, and the PLA_2_ activity was monitored by measuring the fluorescence intensity with the excitation wavelength at 450 nm and the emission at 515 nm on a *LS* 55 fluorescence spectrometer (PerkinElmer).

**GEF activity assay**

GEF activity assay was performed using 1 μM of Rab proteins charged with mant-GDP (Invitrogen) in a reaction buffer containing 0.2 mM GTP, 20 mM Tris-HCl (pH 8.0), 150 mM NaCl and 0.5 mM MgCl_2_, as reported in the literature [2]. Data were collected on the *LS* 55 fluorescence spectrophotometer with the excitation wavelength set to 360 nm and the emission monitored at 440 nm. The catalytic efficiency, *k*_cat_/*K*_M_, was obtained from the slope of fluorescence intensity as previously described [3].

**GTPase activity assay**

GTPase activity was measured according to the protocol provided with the EnzChek phosphate assay kit (Molecular Probes) containing 2-amino-6-mercapto-7-methylpurine riboside substrate, which is converted to ribose 1-phosphate and 2-amino-6-mercapto-7-methylpurine (peak absorbance at 360 nm) by purine nucleoside phosphorylase in the presence of phosphate. Rab5b(1-190) was loaded with GTP in a buffer containing 20 mM Tris (pH 8.0), 150 mM NaCl, 5 mM EDTA, 1 mM DTT and 20 mM GTP at 37 °C for 30 min, and subsequently 5 mM MgCl_2_ was added to neutralize EDTA. The enzyme-substrate mixture was incubated in a reaction buffer containing 5 mM GTP, 50 mM Tris-HCl (pH 7.5), 100 mM NaCl, 1.5 mM MgCl_2_ and 5% glycerol at room temperature for 20 min and mixed with protein samples. The absorbance increase at 360 nm by the phosphorolysis of the substrate was recorded on a Libra S22 ultraviolet/visible spectrophotometer (BioChrom).

**Cell culture and live cell imaging**

HeLa cells and mouse macrophage RAW264.7 cells were purchased from the American Type Culture Collection and cultured in Dulbecco’s Modified Eagle Medium (DMEM) (Gibco-BRL) supplemented with 10% fetal bovine serum (FBS) (Gibco-BRL) at 37 ^o^C. The pEYFP-N1 and pECFP-C1 vectors were used to clone Rab and VipD proteins. Cells were transfected with these vectors using Lipofectamine 2000 (Invitrogen) according to the manufacturer’s instructions. At 16 h after transfection, cells were incubated with Lysotracker Red DND-99 (Molecular Probes) at 50 nM for 30 min. After washing with a phosphate-buffered saline (PBS) buffer, cells were imaged with a Nikon A1RSi confocal microscope (60x CF I Plan Apo oil objective). All of the images were captured and analyzed with the Nikon imaging software (NIS-element AR 64-bit version 3.00, Laboratory Imaging).

**siRNA preparation and transfection**

The siRNAs against Rab5a, Rab5b, Rab5c and Rab22a were designed as reported [4-7] and purchased from Bioneer. The sequences are 5'-^82^GAGTCCGCTGTTGGCAAATCA-3' (against Rab5a), 5'-^95^GAAAGTCAAGCCTGGTATT-3' and 5'-^101^CAAGCCTGGTATTACGTTT-3' (against Rab5b), 5'-^310^ACCAACACAGATACATTTGCA-3' (against Rab5c), 5'-^400^AAGGACTACGCCGACTCTATT-3' and 5'-^481^GAAATTAGTCGAAGAATTC -3' (against Rab22a). A 9-mer *AccuTarget™* siRNA duplex (Bioneer) was used as a negative control. Each siRNA was synthesized as a duplex with UU-overhang, and the antisense strand was chemically phosphorylated. The transfection was performed using Lipofectamine 2000 according to the manufacturer’s instructions.

**RAW264.7 cell culture**

RAW264.7 cells were maintained in DMEM containing 10% FBS, sodium pyruvate, nonessential amino acids, penicillin G (100 IU/mL) and streptomycin (100 μg/mL) (Gibco-BRL). The cells were transfected with the pCDH-CMV vector encoding a VipD protein using Lipofectamine 2000 according to the manufacturer’s instructions. Stable cell lines were generated using a standard selection protocol with 4 μg/mL puromycin (Sigma-Aldrich).

**Immunoblotting and immunoprecipitation**

For immunoblotting, polypeptides were resolved by SDS-polyacrylamide gel electrophoresis and transferred onto a PVDF membrane (Bio-Rad). Immunodetection was achieved using a chemiluminescence reagent (Pierce) and a Fuji Phosphorimager. For immunoprecipitation, the RAW264.7 cells stably expressing VipD proteins were harvested and then lysed in a 0.5% Triton X-100 buffer (Boston BioProduct) supplemented with a complete protease inhibitor cocktail (Roche). After pre-clearing with protein A/G agarose beads for 1 h at 4 °C, whole-cell lysates were used for immunoprecipitation with antibodies. Generally, 1-4 μg of the commercial antibodies was added to 1 mL of the cell lysate, which was then incubated at 4 °C for 8-12 h. After addition of protein A/G agarose beads, incubation was continued for additional 2 h. Immunoprecipitates were extensively washed with a Triton X-100 lysis buffer and eluted with a SDS-PAGE loading buffer by boiling for 5 min. Immunodetection was achieved with anti-Flag, anti-actin, anti-Rab5b and anti-Rabaptin-5 antibodies (Santa Cruz Biotechnology).

**Flow cytometry and** **confocal fluorescence microscopy**

RAW264.7 cells were incubated with 20 μg/mL DQ-Red BSA (Invitrogen) in complete DMEM at 37 ^o^C for 30 min. After washing twice with ice-cold PBS to remove excessive probe, cells were stimulated with LPS (100 ng/mL) for 0-9 hours and harvested and resuspended in PBS containing 4% paraformaldehyde. Red-fluorescent DQ-Red BSA was analyzed by flow cytometry using FACSCanto II (BD Bioscience) and FlowJo software (Treestar, Inc.) or visualized by an Eclipse C1 confocal microscope (Nikon). For immunostaining, RAW264.7 cells were seeded on 12-well culture dishes that contained 18 mm diameter round glass coverslips (105 cells per well). Cells were fixed with 4% paraformaldehyde in PBS for 10 min and permeabilized with 0.25% Triton X-100 in PBS for 15 min before treatment with 10% BSA and 3% FBS for 1 hr. Fixed cells were then stained with primary antibodies, mouse anti-TLR4, rabbit anti-EEA1 and rabbit anti-LAMP-1 (Santa Cruz Biotechnology), overnight at 4 ^o^C. After extensive washing with 0.5% Tween20-added PBS to remove excessive primary antibodies, fixed cells were incubated for 30 min at room temperature with fluorescently labeled secondary antibodies, anti-mouse IgG-TRITC and anti-rabbit IgG-FITC (Molecular Probes), followed by extensive washing with 0.5% Tween20-added PBS twice and with PBS alone once. Cells were imaged with an Eclipse C1 confocal microscope. For phagocytosis analysis, RAW264.7 cells were incubated with mCherry-expressing *E. coli* cells (BL21(DE3) strain) (Novagen) at the multiplicities of infection of 10 at 37 ^o^C for 2 hours. Then the supernatant was replaced with Dulbecco's phosphate-buffered saline (Gibco-BRL) containing gentamicin (400 μg/mL) and kanamycin (200 μg/mL) to kill extracellular bacteria. After 20 min, cells were washed with OPTi-MEM (Life Technologies) five times and the medium was replaced with DMEM containing 10% FBS, 100 μg/mL gentamicin and 100 μg/mL kanamycin. Cells were fixed at indicated time points and imaged with a Nikon A1RSi confocal microscope.

**Macrophage infection assay**

For infection assays, *L. pneumophila* Philadelphia-1 strains were cultured on buffered charcoal yeast extract agar plates supplemented with 0.1 mg/mL thymidine. *L. pneumophila* Δ*flaA*, Δ*vipD*/Δ*flaA* and Δ*vipD* strains were generated by standard homologous recombination using the suicide plasmid pSR47s and confirmed by PCR. Fresh single bacterial colonies were streaked onto culture plates two day before infection. Bacteria were scraped off, diluted in sterile water and added to BMDM, U937 and A549 cells. Infection was facilitated by centrifugation at 300g for 10 min. *L. pneumophila*-infected cells were analyzed by immunofluorescence microscopy similarly with RAW264.7 cells using anti-human Rab5b, anti-human Rab1b (Santa Cruz Biotechnology), anti-*L. pneumophila* (kindly provided by Dr. Ralph R. Isberg), anti-EEA1, anti-Rabaptin-5, anti-HA (Covance), Alexa Fluor 488/568-conjugated secondary antibodies (Life Technologies) and 4'-6-Diamidino-2-phenylindole (DAPI) for staining nucleus.

**Supplementary References**

1. Shen A, Lupardus PJ, Morell M, Ponder EL, Sadaghiani AM, et al. (2009) Simplified, enhanced protein purification using an inducible, autoprocessing enzyme tag. PLoS One 4: e8119.

2. Murata T, Delprato A, Ingmundson A, Toomre DK, Lambright DG, et al. (2006) The *Legionella pneumophila* effector protein DrrA is a Rab1 guanine nucleotide-exchange factor. Nat Cell Biol 8: 971-977.

3. Delprato A, Merithew E, Lambright DG (2004) Structure, exchange determinants, and family-wide rab specificity of the tandem helical bundle and Vps9 domains of Rabex-5. Cell 118: 607-617.

4. Magadan JG, Barbieri MA, Mesa R, Stahl PD, Mayorga LS (2006) Rab22a regulates the sorting of transferrin to recycling endosomes. Mol Cell Biol 26: 2595-2614.

5. Chen PI, Kong C, Su X, Stahl PD (2009) Rab5 isoforms differentially regulate the trafficking and degradation of epidermal growth factor receptors. J Biol Chem 284: 30328-30338.

6. Weigert R, Yeung AC, Li J, Donaldson JG (2004) Rab22a regulates the recycling of membrane proteins internalized independently of clathrin. Mol Biol Cell 15: 3758-3770.

7. Huang F, Khvorova A, Marshall W, Sorkin A (2004) Analysis of clathrin-mediated endocytosis of epidermal growth factor receptor by RNA interference. J Biol Chem 279: 16657-16661.
